# Characterization of *Entamoeba histolytica* adenosine 5′-phosphosulfate (APS) kinase; validation as a target and provision of leads for the development of new drugs against amoebiasis

**DOI:** 10.1371/journal.pntd.0007633

**Published:** 2019-08-19

**Authors:** Fumika Mi-ichi, Takeshi Ishikawa, Vo Kha Tam, Sharmina Deloer, Shinjiro Hamano, Tsuyoshi Hamada, Hiroki Yoshida

**Affiliations:** 1 Division of Molecular and Cellular Immunoscience, Department of Biomolecular Sciences, Faculty of Medicine, Saga University, Nabeshima, Saga, Japan; 2 Department of Molecular Microbiology and Immunology, Graduate School of Biomedical Sciences, Nagasaki University, Sakamoto, Nagasaki, Japan; 3 Department of Parasitology, Institute of Tropical Medicine (NEKKEN), Nagasaki University, Sakamoto, Nagasaki, Japan; 4 Nagasaki Advanced Computing Center, Nagasaki University, Bunkyo-machi, Nagasaki, Japan; Jawaharlal Nehru University, INDIA

## Abstract

**Background:**

Amoebiasis, caused by *Entamoeba histolytica* infection, is a global public health problem. However, available drugs to treat amoebiasis are currently limited, and no effective vaccine exists. Therefore, development of new preventive measures against amoebiasis is urgently needed.

**Methodology/Principal findings:**

Here, to develop new drugs against amoebiasis, we focused on *E*. *histolytica* adenosine 5′-phosphosulfate kinase (EhAPSK), an essential enzyme in *Entamoeba* sulfolipid metabolism. Fatty alcohol disulfates and cholesteryl sulfate, sulfolipids synthesized in *Entamoeba*, play important roles in trophozoite proliferation and cyst formation. These processes are closely associated with clinical manifestation and severe pathogenesis of amoebiasis and with disease transmission, respectively. We validated a combination approach of *in silico* molecular docking analysis and an *in vitro* enzyme activity assay for large scale screening. Docking simulation ranked the binding free energy between a homology modeling structure of EhAPSK and 400 compounds. The 400 compounds were also screened by a 96-well plate-based *in vitro* APSK activity assay. Among fifteen compounds identified as EhAPSK inhibitors by the *in vitro* system, six were ranked by the *in silico* analysis as having high affinity toward EhAPSK. Furthermore, 2-(3-fluorophenoxy)-N-[4-(2-pyridyl)thiazol-2-yl]-acetamide, 3-phenyl-N-[4-(2-pyridyl)thiazol-2-yl]-imidazole-4-carboxamide, and auranofin, which were identified as EhAPSK inhibitors by both *in silico* and *in vitro* analyses, halted not only *Entamoeba* trophozoite proliferation but also cyst formation. These three compounds also dose-dependently impaired the synthesis of sulfolipids in *E*. *histolytica*.

**Conclusions/Significance:**

Hence, the combined approach of *in silico* and *in vitro*-based EhAPSK analyses identified compounds that can be evaluated for their effects on *Entamoeba*. This can provide leads for the development of new anti-amoebic and amoebiasis transmission-blocking drugs. This strategy can also be applied to identify specific APSK inhibitors, which will benefit research into sulfur metabolism and the ubiquitous pathway terminally synthesizing essential sulfur-containing biomolecules.

## Author summary

Amoebiasis is a parasitic disease caused by *Entamoeba histolytica* that is an important health problem worldwide because of high morbidity and mortality rates. However, clinical options are inadequate; therefore, developing new preventive measures, such as anti-amoebic drugs, is urgently needed. In general, for the development of new drugs, the identification of appropriate leads and targets is a prerequisite. Here, to develop new drugs against amoebiasis, we focused on *E*. *histolytica* adenosine 5′-phosphosulfate kinase (EhAPSK), an essential enzyme in sulfur metabolism. An EhAPSK-based combination approach of computer-based *in silico* and laboratory-based *in vitro* analyses enabled us to screen 400 chemicals, from which we identified 15 that inhibit EhAPSK activity. Furthermore, among them, three compounds halted biological processes in *Entamoeba* that are closely associated with the clinical manifestation and pathogenesis of amoebiasis and with disease transmission. Hence, this study provides leads as well as a target for the development of new drugs against amoebiasis. This study also provides a basis to identify inhibitors for use in the study of sulfur metabolism, an important topic in general biochemistry and physiology.

## Introduction

Amoebiasis, a parasitic disease, causes high morbidity and mortality; approximately 50 million cases of disease and 40,000–70,000 deaths annually [1]. Typical symptoms of this disease include diarrhea, dysentery, fever, and abdominal pains, which are diagnosed as intestinal manifestations. Patients sometimes develop extra-intestinal amoebiasis with amoebic liver abscess being most commonly diagnosed. Together with these obvious clinical cases, a high occurrence of asymptomatic patients who unconsciously spread the disease makes amoebiasis a global public health problem. Therefore, not only symptomatic but also asymptomatic patients need appropriate medical treatments [2]. However, clinical options are currently inadequate; available drugs are limited, and no effective vaccine exists [3, 4]. Therefore, the development of new preventive measures against amoebiasis is urgently needed.

This parasitic disease is caused by *Entamoeba histolytica*. This protozoan parasite maintains a life cycle of a proliferative trophozoite and dormant cyst stages. Trophozoites differentiate into cysts, the only form able to transmit amoebiasis, whereas cysts hatch into trophozoites, the form able to proliferate and invade the intestinal mucosal tissue. These two capabilities of *E*. *histolytica* trophozoites are closely associated with the clinical manifestations and severe pathogenesis of amoebiasis [5–7]. Therefore, killing *E*. *histolytica* trophozoites cures amoebiasis patients, whereas halting cyst formation blocks the disease transmission. A combination strategy against *E*. *histolytica* trophozoites and cysts can lead to eradication of the global burden of amoebiasis [5]. To attain this ultimate goal, developing new amoebiasis transmission-blocking as well as anti-amoebic drugs is essential. For this objective, targeting *E*. *histolytica* sulfur metabolism is ideal because it has pleiotropic roles in the maintenance of the parasite’s life cycle via its terminal products, sulfolipids [8, 9]. The overall scheme and physiological roles of this metabolism have recently been demonstrated [8–12]. Briefly, sulfate in the external milieu is trafficked via at least two transporters to mitosomes, a type of highly diversified mitochondrion, and is then activated by two sequential reactions catalyzed by ATP sulfurylase (AS) and adenosine 5′-phosphosulfate (APS) kinase (APSK), to produce 3′-phosphoadenosine 5′-phosphosulfate (PAPS) [10, 11]. Subsequently, PAPS is transported from mitosomes to the cytosol by members of the mitochondrial carrier family and is utilized by sulfotransferases to synthesize sulfolipids, the major terminal metabolites [8–10, 12]. Fatty alcohol disulfates play an important role in *E*. *histolytica* trophozoite proliferation whereas cholesteryl sulfate is a key molecule in the regulation of *Entamoeba* cyst formation [8, 9]. Therefore, abolishing the production of these sulfolipids in *E*. *histolytica* can arrest trophozoite proliferation and also halt cyst formation. This will block the transmission of as well as cure, amoebiasis.

To inhibit the production of terminal metabolites, enzymes solely functioning at earlier steps in the pertinent metabolic pathway are suitable targets; for instance, AS and APSK in *E*. *histolytica* sulfur metabolism. Knocking down the single genes encoding either *E*. *histolytica* AS (EhAS) or APSK (EhAPSK) retarded the proliferation of trophozoites [11]. Furthermore, *in vitro* treatment of *Entamoeba invadens* culture by an inhibitor, the putative target of which is AS, reduced the number of cysts formed [8]; in *Entamoeba* encystation studies, the *in vitro* culture of *E*. *invadens*, a reptilian parasite, and not that of *E*. *histolytica*, has been adopted as a model system [5, 13]. It is worth mentioning that the limited availability of inhibitors specific for AS and APSK is a major drawback in sulfur metabolism research, not only in *Entamoeba* but also in other organisms; sulfur metabolism is a ubiquitous pathway that terminally synthesizes a variety of sulfur-containing biomolecules crucial in living organisms, such as sulfolipids, sulfated polysaccharides, cysteine, methionine, and Fe-S cluster [14, 15].

As well as appropriate targets, providing desirable leads is essential for the development of new drugs; therefore, a variety of methods for screening large numbers of compounds have been undertaken as the prerequisite step. For instance, high through-put *in vitro* assays using recombinant enzymes [16, 17], wild-type cells [18–20], or an engineered cell [20] have been used. However, screening a huge number of compounds, *e*.*g*., more than a million, is technically demanding. To compensate for this limitation of *in vitro* screening, *in silico* docking simulation is effective; the docking simulation rapidly predicts the binding free energy between a small molecule and a target protein using empirical energy functions, from which we can select potential leads for drug development from a large chemical library [16, 21]. Hence, *in silico* docking simulation has been performed as the primary screen in many drug discovery projects [22, 23].

Here, in aiming to develop new anti-amoebic and amoebiasis transmission-blocking drugs, we focused on EhAPSK and examined whether it can be a target. We assessed the validity of an EhAPSK-based combination approach comprising an *in silico* molecular docking analysis and *in vitro* activity assay, for large scale screening to provide desirable leads. We also characterized three compounds, identified from screening the 400 chemicals in the Pathogen Box from the Medicines for Malaria Venture (MMV; https://www.pathogenbox.org/), to make a causal link between inhibition of EhAPSK activity and halting biological processes essential for maintenance of the *Entamoeba* life cycle. For this analysis, we focused on sulfolipids, the terminal products in the metabolic pathway in which EhAPSK is involved.

## Methods

### Materials

APS (>85% purity) and phosphoenolpyruvate (PEP) (>99% purity) were purchased from Sigma-Aldrich (St. Louis, MO, USA) and dissolved in deionized water at 5.5 mM and 400 mM, respectively, as stock solutions. NADH (>95% purity) was from Sigma-Aldrich and dissolved in 10 mM NaOH to make a 100 mM stock solution. Na_2_ATP (98% purity) was purchased from Wako (Osaka, Japan). A premixed stock solution containing 100 mM Na_2_ATP and 50 mM MgC1_2_ was prepared using 0.24 M Tris-free base. Nuclease P1 (NP1) (catalogue No. 145–08221) was from Wako and dissolved in deionized water at 1 unit/μl as a stock solution. Pyruvate kinase (PK) (catalogue No. 301–50713) and lactate dehydrogenase (LDH) (catalogue No. 300–52721) were from Wako and used to prepare a premixed stock solution [0.7 units/μl PK, 1 unit/μl LDH, 100 mM KCl, 50% glycerol, and 0.1 mM EDTA in 10 mM Hepes-KOH (pH 7.2)]. All stock solutions were stored at -30°C before use.

The storage conditions of the 400 compounds in the Pathogen Box, obtained from MMV, and the supplier and storage condition of auranofin (>98% purity) (which is E-H-05 in the Pathogen Box) were described in [18]. 2-(3-fluorophenoxy)-N-[4-(2-pyridyl)thiazol-2-yl]-acetamide (>96% purity) and 3-phenyl-N-[4-(2-pyridyl)thiazol-2-yl]-imidazole-4-carboxamide (>96% purity), which are A-D-11 and A-H-11, respectively, in the Pathogen Box, were purchased from Enamine Ltd. (Kiev, Ukraine), dissolved in dimethyl sulfoxide (DMSO) at 10 mM as stock solutions and stored at -30°C in 40 μL aliquots.

### Multiple sequence alignment of APSK and proteins containing an APSK domain

Homologues of the EhAPSK APSK domain (Ile^294^—Lys^477^) were retrieved from UniProtKB (https://www.uniprot.org/help/uniprotkb) based on the criteria that the origin is ‘different organism’ and its tertiary structure is ‘unravelled’. A multiple sequence alignment of the EhAPSK APSK domain and the nine retrieved proteins (a whole protein or the APSK domain of a protein) was then made using MUSCLE [24].

### Homology modeling of the APSK domain structure of EhAPSK

Twenty model structures of the EhAPSK APSK domain [Ile^294^—Lys^477^; AmoebaDB (http://amoebadb.org/amoeba/) ID, EHI_179080] were generated from each of three templates [RCSB PDB (http://www.rcsb.org/) IDs, 3UIE, 4FXP, and 2GKS], from which co-crystalized substrates (APS and ATP-analog) and a product (ADP) were removed, using Modeller 9.15 [25]. Among the twenty model structures generated, the most fitted structure, which showed the highest values of the MODELLER objective function (molpdf; [26]) and discrete optimized protein energy (DOPE; [27]), was selected. In total, therefore, three distinct homology modeling structures of the EhAPSK APSK domain were predicted and annotated as EhAPSK structure-A, -B, and -C based on the template used (3UIE, 4FXP, and 2GKS, respectively). Root mean square deviations (RMSDs) of main-chain atoms between all combinations of the three predicted structures were calculated by visual molecular dynamics (VMD; [28]).

### Molecular docking analysis

AutoDock 4.2 [29], a standard docking simulation software based on a genetic algorithm, was used to set a cubic space that covers the binding site of APS as a search region, *i*.*e*., 26.3 × 26.3 × 26.3 Å for EhAPSK structures A and B and 22.5 × 22.5 × 22.5 Å for structure C. Then, docking simulation between EhAPSK structures A, B, and C and each compound (the three-dimensional structure of which was provided from SMILES notations using Open Babel [30]), was performed. Twenty individual calculations were run with the genetic algorithm (“ga_run” = 20), and in each run, 10^7^ energy calculations were performed (“ga_num_evals” = 10^7^). Degrees of freedom of the target protein, EhAPSK, were fixed during the docking simulation. Because AutoDock 4.2 does not include a parameter for boron, the boron atom-containing compounds in the Pathogen Box, such as B-A-03, B-B-05, and B-E-05, were analyzed using a parameter for carbon, *i*.*e*., parameter for atom type “C”, at the position of the boron atom. Neither is there a parameter for Au (gold); therefore, the Au-containing compound, E-H-05, was analyzed in an Au-dissociated form.

### Production and purification of recombinant EhAPSK (rEhAPSK) and recombinant *Homo sapiens* APSK (rHsAPSK)

All the primers used for plasmid constructions are listed in S1 Table. To produce rEhAPSK in *E*. *coli*, a DNA fragment encoding the whole protein consisting of nonfunctional AS-like and catalytic APSK domains (AmoebaDB ID, EHI_179080) was PCR-amplified from *E*. *histolytica* (HM-1:IMSS cl6) cDNAs. The amplicon showing the expected size was purified, digested with XhoI/PstI, and inserted into the corresponding sites of pCold-I^™^ (Takara, Kyoto, Japan), an expression plasmid designed for histidine (His)-tagged recombinant protein production. An appropriate plasmid was selected by sequencing the inserted fragment, and the obtained plasmid was designated as pCold-I-EhAPSK.

To determine the representative rHsAPSK, three plasmids, pCold-I-HsAPSK1 and -2, and -HsPAPSS1, were likewise constructed as described above, except that the regions encoding the APSK domains of the corresponding bifunctional *H*. *sapiens* PAPS synthase 1 and -2 (HsPAPSS1 and HsPAPSS2), Ala^25^—Pro^227^ and Ser^15^—Pro^217^ [GenBank (https://www.ncbi.nlm.nih.gov/genbank/) Accession No: NP_005434 and NP_004661, respectively], and the full-length HsPAPSS1 were individually amplified from THP1 cell line cDNAs. XhoI/PstI or NdeI/SalI sites of pCold-I^™^ (Takara) were used to insert each of the three appropriate amplicons.

To prepare an unrelated protein control, a His-tagged recombinant *E*. *histolytica* sulfatase 2 (rEhSF2) (AmoebaDB ID, EHI_198980), pCold-I-EhSF2, similar to pCold-I-EhAPSK, was constructed using an appropriate primer set. BamHI/SalI sites of pCold-I^™^ (Takara) were used to insert the amplicon.

Single colonies from the glycerol stocks of *E*. *coli* [BL21(DE3) Singles^™^ Competent Cells (Merck, Darmstadt, Germany)] transformants harboring each of the above plasmids were cultivated in 3 mL LB medium containing 1 μg/mL ampicillin at 37°C for 4–6 h. Each pre-culture was then transferred into 100 mL LB medium containing 1 μg/mL ampicillin, and the resulting main culture was incubated at 37°C with rigorous shaking. When the culture reached OD_600_ 0.4–0.5, the culture flask was stood at 15°C for 30 min without shaking. After addition of IPTG to a final concentration of 1 mM, the culture was restarted with shaking at 15°C for 24 h. Subsequently, cells were harvested by centrifugation at 4400 g for 5 min at 4°C, and the cell pellet was stored at -80°C until use.

The cell pellet prepared from the 100 mL culture was completely resuspended in 20 mL lysis buffer [50 mM Tris/HCl (pH 8.0) containing 300 mM NaCl, 20 mM imidazole, 10% glycerol (vol/vol), 1% Triton X-100 (vol/vol), and 100 μg/mL lysozyme]. After adding 20 μL Benzonase^™^ Nuclease (25 U/μL) [>99% purity, Merck (Kenilworth, NJ, USA)] and 100 μL 1 M MgCl_2_ (final concentrations were 25 U/ml and 5 mM, respectively), the mixture was incubated at 4°C for 15 min with gentle mixing using a rotator. Then the mixture was sonicated in an ice bath at five cycles of 30 sec with 30 sec intervals, followed by centrifugation at 12,000 g for 30 min at 4°C.

The supernatant collected as a crude enzyme solution was purified by affinity column chromatography using a prepacked nickel bound resin, His GraviTrap^™^, from GE Healthcare Life Sciences (Buckinghamshire, UK) according to the manufacturer’s instructions. In detail, ~20 mL crude enzyme solution was applied to the prepacked column (1 mL bed volume) equilibrated with wash buffer [50 mM Tris/HCl (pH 8.0) containing 300 mM NaCl and 20 mM imidazole]. After washing the column with eight bed volumes of wash buffer, the bound samples were eluted by a stepwise increase of imidazole concentration at 50, 100, 250, and 500 mM using 10 bed volumes of elution buffer [50 mM Tris/HCl (pH 8.0) containing 300 mM NaCl] for each concentration of imidazole. Each ~0.5 mL was manually collected as a fraction during column chromatography. All different His-tagged recombinant proteins synthesized in *E*. *coli* were enriched in three fractions eluted at 250 mM imidazole. These three fractions were pooled and then stored at 4°C before use either as purified recombinant APSK solution (rEhAPSK or rHsAPSK) or as a mock control (rEhSF2) because the activities of both rEhAPSK and rHsAPSK were relatively stable at 4°C, but rEhAPSK activity was labile to freeze-thaw because of aggregation; the specific activity of rEhAPSK after storage for 3 months at 4°C was ~25% relative to that of freshly purified samples whereas that of rHsAPSK after one month storage at 4°C was ~20%. In contrast, rEhAPSK activity was almost entirely absent after storage for 3 days or more at -30°C, when precipitates became visible. Note that all purified enzyme solutions were used within a week.

To prepare denatured APSKs, 30 μL of purified rEhAPSK or rHsAPSK solutions were incubated in a 1.5 mL tube at 95°C for 10 min using a heating block. An appropriate volume of the suspension was then used; for instance, 0.5 μg purified rEhAPSK or 3.5 μg purified rHsAPSK.

### Establishment of the 96-well plate-based APSK activity assay

A coupling assay to measure APSK activity was performed as described [31, 32] except a 96-well plate was used. Briefly, each well contained 100 μL reaction mixture, which consisted of 90 μL coupling solution and 10 μL of either compound solution (see below) or 10% DMSO. Enzymatic reaction was started upon addition of 10 μL of 110 μM APS solution and immediately monitored by absorbance change at 340 nm with time using either an EnVision or ARVO plate reader (PerkinElmer, Waltham, MA, USA). The coupling solution was 100 mM Tris/HCl (pH 8.0), 1 mM MgCl_2_, 1 mM KCl, 5 mM Mg_2_ATP, 1 mM NADH, 1 mM PEP, 1 U NP1, 3.5 U PK, 5.0 U LDH, and APSK (≤3 μL). Note that the effect of elution buffer used for enriching recombinant proteins in column chromatography on APSK activity was negligible at ≤5 μL. As controls, in place of purified APSK solution (rEhAPSK or rHsAPSK), 3 μL of a blank [50 mM Tris/HCl buffer (pH 8.0)], a solvent (elution buffer), a mock (rEhSF2), or denatured APSK suspensions obtained by heat-treatment, were added to the coupling solution. The activity of each sample was calculated after removing the baseline value of the blank control.

When needed, a set of 400 compounds, which were adjusted to a concentration of 1 mM each as described in [18], was thawed from -30°C storage, and then used after appropriate dilution.

For the inhibition assay, 2 μL of each stock solution, which were distributed in a 96-well plate, were transferred to a new plate to make a replicate. After adding 18 μL deionized water to each well and mixing, 10 μL of each diluted solution (100 μM compound solution) was again transferred into a new 96-well plate to make a replicate. Then 90 μL of the coupling solution prepared above was dispensed into each well containing the previously added 10 μL of 100 μM compound solution. As a solvent control, 10% DMSO solution (vol/vol-deionized water) was used in place of compound solution. The final concentrations of each compound and DMSO in a well were 10 μM and 1% (vol/vol), respectively. The assays were performed independently three times, and data are shown as the mean with SD.

To determine IC_50_ values, the 1 mM stock solution of each compound to be tested was serially diluted by 3-fold decrements with DMSO. One millimolar stock solutions used were either thawed in a replicate (96-well plate) prepared in [18] (A-C-05, A-F-04, A-F-07, B-B-05, B-C-02, B-C-08, B-D-03, C-C-06, C-F-03, C-F-06, D-E-10, and E-G-10 in the Pathogen Box) or diluted with DMSO from 10 mM stock solutions of commercially available compounds (A-D-11, A-H-11, and E-H-05 in the Pathogen Box) in DMSO. Subsequently, 3.4 μL of the 1 mM and each diluted stock solution were dispensed into wells of a 96-well culture plate, containing 30.6 μL deionized water and mixed well by pipetting. Ten microliters of all resulting solutions were then dispensed into wells of a 96-well plate to make triplicates and then used as described for the above inhibition assay. 10% DMSO solution was used as an inhibitor-free control. Note that the final DMSO concentration in any well was 1% (vol/vol).

In all assays, prepared mixtures in a plate were mixed on a vibrating table equipped with either an EnVision or ARVO plate reader (PerkinElmer) before starting the reaction. Immediately after addition of APS solution and mixing again, absorbance change at 340 nm was monitored every 2 min.

### *E*. *histolytica* trophozoite proliferation and *E*. *invadens* cyst formation assays, metabolic labeling, and morphological analysis

*E*. *histolytica* trophozoite proliferation and *E*. *invadens* cyst formation assays were performed essentially as described in [18] except that in addition to 0–72 and 48–72 h cyst formation assays, 0–96 and 0–120 h, and 48–96 and 48–120 h assays were also performed.

Metabolic labeling of *E*. *histolytica* trophozoites by [^35^S]-labeled sulfate was performed essentially as described previously [9] with slight modification. In detail, 2.5×10^5^ *E*. *histolytica* trophozoites were treated with various concentrations of each compound (A-D-11, A-H-11, or E-H-05 in the Pathogen Box) in 0.75 ml BI-S-33 medium at 37°C for 6 h under anaerobic conditions using Anaerocult A (Merck). Each 10 mM stock solution (in DMSO) of the three commercially sourced compounds was serially diluted by 2-fold decrements with DMSO. The resulting diluted solutions were then added to 0.75 ml cell suspension in BI-S-33 medium at 1/100 (vol/vol). The final concentrations of A-D-11 and A-H-11 in the medium were 6.25, 12.5, 25, 50, and 100 μM, whereas those of E-H-05 were 0.5, 1, 2, 4, and 8 μM. Note that DMSO was used as a solvent control at 1%, which was the DMSO concentration in all samples analyzed. [^35^S]-labeled sulfate (45 μCi) was added 4 h after the start of treatments and labeled cells were harvested at 6 h. Lipids were extracted with 0.5 ml methanol and were separated by high-performance thin layer chromatography (HPTLC) (chloroform/methanol/28% (wt/wt) ammonium hydroxide [65:35:8; vol/vol/vol]) on a silica gel plate (no. 1.05641.0001; Merck). Each spot on the TLC plates was quantified using a Fuji imaging analyzer and Multi Gauge 2.2 software (FLA-7000; Fujifilm, Japan). The total cell protein was estimated using a Pierce BCA protein assay kit (Thermo Scientific, Rockford, IL, USA).

*E*. *histolytica* trophozoites were treated with A-D-11, A-H-11, E-H-05, or 1% DMSO (solvent control) for 6 h as described above except for the omission of the [^35^S]-labeling step. Cell morphology was then observed by phase contrast microscopy using a Primovert microscope (Carl Zeiss, Germany) with a COMS camera (E3CMOS12000KPA, ToupTek, China). The concentrations of A-D-11 and A-H-11 used were 6.25, 12.5, 25, 50 and 100 μM, whereas those of E-H-05 were 0.5, 1, 2, 4, and 8 μM. Cell images were captured using ToupView software (ToupTek).

### Cytotoxicity assay

Human foreskin fibroblast (HFF) cells (purchased from ATCC) were routinely maintained in DMEM supplemented with 10% fetal bovine serum, 100 units/ml penicillin, 100 mg/ml streptomycin and 1% GlutaMAX^™^-I (Thermo Fisher Scientific, Waltham, MA). A WST-1-based cytotoxicity assay was performed as previously described [33]. In detail, HFF cells were plated at 1.0×10^4^ cells/100 μL medium in each well of a 96-well culture plate, and incubated overnight at 37°C in a 5% CO_2_ atmosphere to allow cells to adhere to the wells. Cells were then exposed to 10 μM of each test compound, 100 μM of A-D-11 or A-H-11, or various concentrations of E-H-05 (auranofin) for 48 h. Numbers of viable cells were then assessed using the Premix WST-1 Cell Proliferation Assay System (Takara). Either 1 mM stock solutions (A-C-05, A-D-11, A-F-04, A-F-07, A-H-11, B-B-05, B-C-02, B-C-08, B-D-03, C-C-06, C-F-03, C-F-06, D-E-10, E-G-10, and E-H-05 in the Pathogen Box), which were stored in a replicate 96-well plate prepared as described in [18], or 10 mM stock solutions of commercially available compounds (A-D-11 and A-H-11 in the Pathogen Box) in DMSO were added to each well at 1/100 (vol/vol). Solutions prepared by 2-fold serial dilution of the commercially available 10 mM auranofin stock in DMSO (E-H-05 in the Pathogen Box) were likewise added to final concentrations of 0.625, 1.25, 2.5, 5, and 10 μM. DMSO was added at 1% as a negative control whereas ionomycin was added at 30 μM as a positive control. As a blank, the above culture medium was used in place of the solutions added.

## Results

### *In silico* molecular docking analysis between homology modeling structures of EhAPSK and 400 compounds of the MMV Pathogen Box

**Fig 1:**
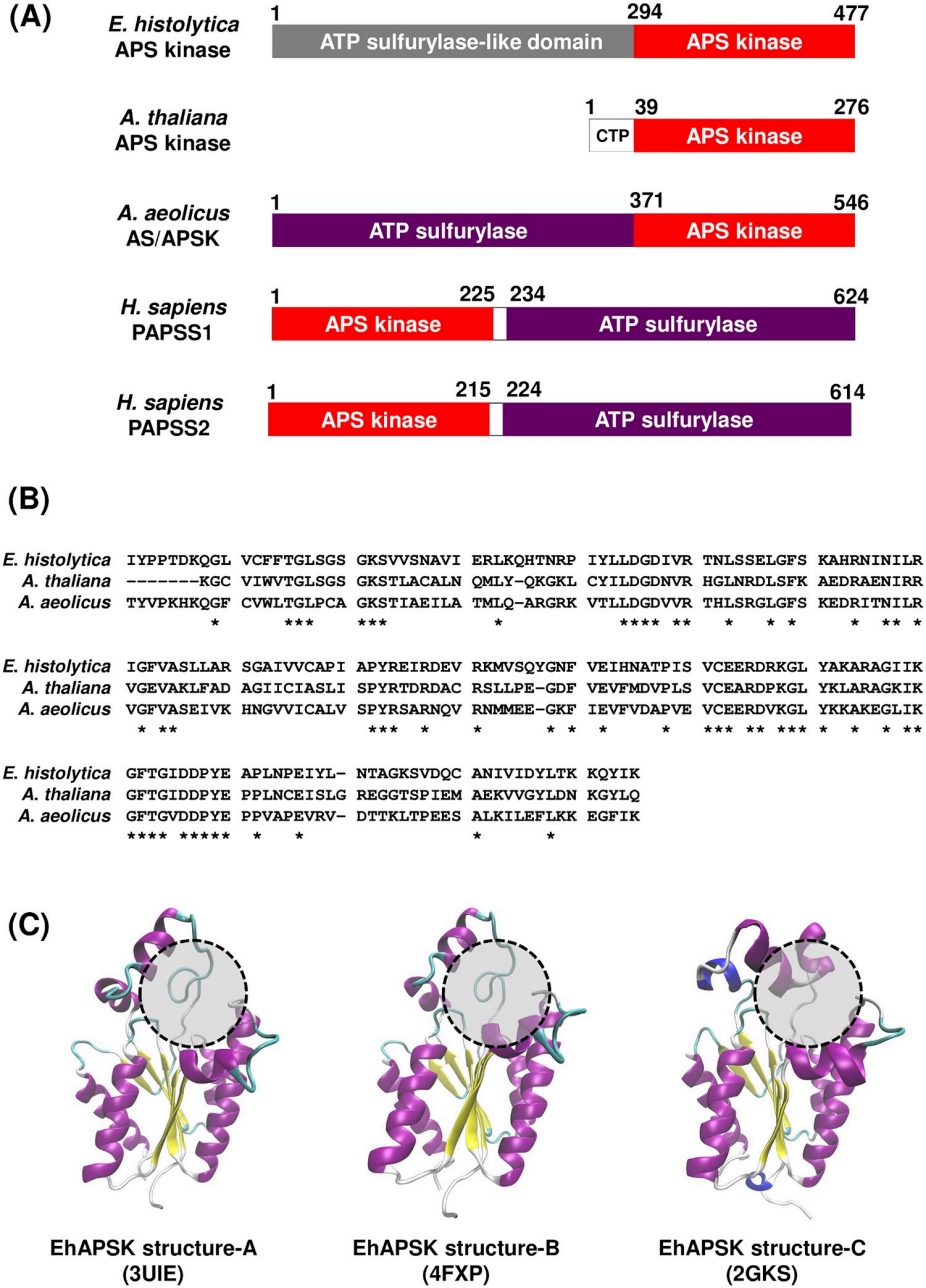
Prediction of the tertiary structure of the EhAPSK APSK domain. (A) Schematic illustration for the arrangement of the APS kinase and ATP sulfurylase domains. Red and purple represent APS kinase and ATP sulfurylase domains, respectively. CTP, chloroplast targeting peptides. (B) Sequence alignments of APSK domains from *E*. *histolytica* APSK, *A*. *thaliana* APSK, and *A*. *aeolicus* PAPSS, which were extracted from S1 Fig. The amino acid residues conserved among these three proteins are indicated by *. (C) Homology modeling structures of the EhAPSK APSK domain. Three different homology modeling structures, EhAPSK structure-A, -B, and -C, are shown. PDB IDs shown inside parentheses indicate the template tertiary structure used for generating each structure. The APS binding site of each structure is shown by a dashed circle.

Docking simulation between a target protein and large numbers of molecules is an effective method to provide potential leads for development of new drugs. The effectiveness becomes substantial when the tertiary structure of a target protein is known [22, 23]. However, no reliable tertiary structure of EhAPSK was available because neither x-ray crystallography nor nuclear magnetic resonance spectroscopy of EhAPSK has been reported. We, therefore, predicted its tertiary structure by homology modeling for which an appropriate template is required. EhAPSK is composed of nonfunctional AS-like and catalytic APSK domains (Fig 1A, AmoebaDB ID, EHI_179080; UniProtKB ID, C4LUW9) [10]. To select the template, a multiple sequence alignment of the EhAPSK APSK domain (Ile^294^—Lys^477^) and nine homologs from different organisms, for which tertiary structures were reported, was made using MUSCLE (S1 Fig and Table 1; [24]). The protein with the most identity to the EhAPSK APSK domain was *Thiobacillus denitrificans* APSK (UniProtKB ID, Q3SM86) (amino acid sequence identity, 54.4%; S1 Fig and Table 1); however, its crystal structure did not include the binding site of a substrate (APS) [RCSB PDB ID, 3CR8; [34]]. Structural information on the binding pockets for substrate and product was provided by x-ray crystallography of *Arabidopsis thaliana* APSK (AtAPSK) (UniProtKB ID, Q43295), which was the second most identical protein (47.83%), and by that of *Aquifex aeolicus* PAPS synthase (AaPAPSS; UniProtKB ID, O67174), the APSK domain of which showed the third highest identity (46.20%) (Fig 1A and 1B and S1 Fig; Table 1). The AtAPSK structures were resolved from two crystal forms (PDB IDs, 3UIE and 4FXP); one form was a complex structure of the enzyme, with two substrates, APS, and an ATP analog, phosphoaminophosphonic acid-adenylate ester (3UIE; [35]). The other form was a complex structure of enzyme and only one substrate (APS) (4FXP; [36]). The complex structure of AaPAPSS enzyme and a product (ADP) was shown by x-ray crystallography (PDB ID, 2GKS; [37]). It should be mentioned that AtAPSK has only the APSK domain and forms a homodimer whereas AaPAPSS is composed of functional AS and APSK domains and forms a homohexamer (Fig 1A) [36, 37]. Collectively, based on the structural information together with sufficiently high amino acid sequence identities, the above three x-ray structures (3UIE, 4FXP, and 2GKS) were chosen as appropriate templates for homology modeling the structure of the EhAPSK APSK domain (Ile^294^—Lys^477^). An independent structure was then generated from the each template (3UIE, 4FXP, and 2GKS) using Modeller9.15 [25], and the three predicted structures were named EhAPSK structure-A, -B, and -C, respectively (Fig 1C). The RMSD of main-chain atoms between EhAPSK structure-A and -B, both of which were generated from AtAPSK crystal templates (3UIE and 4FXP, respectively), was 0.42 Å, whereas those between EhAPSK structure-C, which was generated from the AaPAPSS crystal template (2GKS), and -A or -B were 2.83 or 2.82 Å, respectively. This result indicates that the APS binding sites (marked by dashed circles in Fig 1C) of EhAPSK structure-A and -B were very similar, but different from that of EhAPSK structure-C (Fig 1C).

**Table 1:** Amino acid sequence identity between the APSK domain of *E*. *histolytica* and the APSK domain of nine homologues. AS, ATP sulfurylase; APSK, APS kinase; PAPSS, PAPS synthase.

Species	Protein	UniProt ID	Identity (%)
***Thiobacillus denitrificans***	**AS-like domain/APSK**	**Q3SM86**	**54.4**
***Arabidopsis thaliana***	**APSK**	**Q43295**	**46.7**
***Aquifex aeolicus***	**AS/APSK**	**O67174**	**46.2**
***Synechocystis* sp. PCC 6803**	**APSK**	**P72940**	**45.7**
***Mycobacterium tuberculosis***	**AS/APSK**	**P9WNM5**	**44.0**
***Penicillium chrysogenum***	**APSK**	**Q12657**	**43.5**
***Homo sapiens***	**PAPSS2**	**O95340**	**41.3**
***Homo sapiens***	**PAPSS1**	**O43252**	**40.8**
***Aeropyrum pernix***	**APSK**	**Q9YCR6**	**35.3**

Subsequently, docking simulation was performed to evaluate the binding free energies between each of EhAPSK structure-A, -B, and -C and each of 400 compounds possessing various scaffolds, in the MMV Pathogen Box, using AutoDock 4.2 [29] (S2 Table). As a search region, a cubic space including the binding site of APS was selected. In spite of structural similarity between EhAPSK structure-A and B, structure-B mostly bound to different compounds at lower calculated free energies than structure-A. We mainly attribute this to differences in the spatial position of amino acid residue side chains critical for binding to compounds (S2 Fig); for instance, side chains of Arg^420^ and Lys^421^ embedded in the cavity of EhAPSK structure-B increased electrostatic interaction between the enzyme and compounds, giving lower free binding energies than EhAPSK structure-A, the corresponding amino acids of which protruded from the cavity. Consequently, a single compound, such as A-D-11, showed different binding patterns for each of the two EhAPSK structures (S3 Fig).

### *In vitro* APSK activity assay-based screening of the 400 Pathogen Box compounds

**Fig 2:**
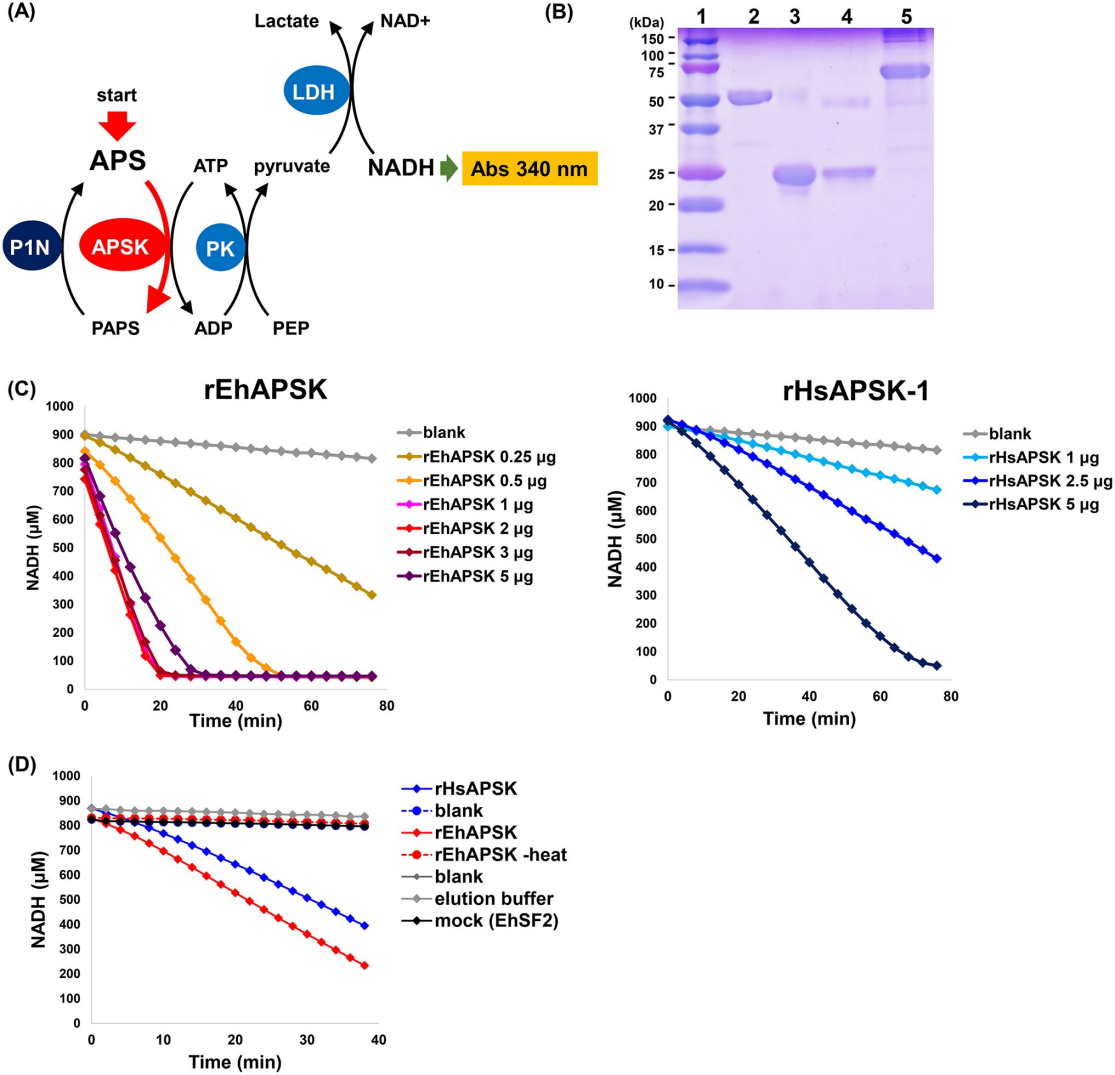
Establishment of the *in vitro* 96-well plate-based APSK activity assay. (A) Schematic illustration of the coupling assay to measure APSK activity. APS, adenosine 5′-phosphosulfate; APSK, APS kinase; PAPS, 3′-phosphoadenosine 5′-phosphosulfate; NP1, nuclease P1; PK, pyruvate kinase; PEP, phosphoenolpyruvate; and LDH, lactate dehydrogenase. (B) SDS-PAGE of the affinity-purified recombinant proteins. Lane 1, molecular weight markers; lane 2, rEhAPSK; lane 3, rHsAPSK (recombinant APSK domain of HsPAPSS1); lane 4, recombinant APSK domain of HsPAPSS2; and lane 5, recombinant HsPAPSS1 (full-length HsPAPSS1). (C) Dose-dependent effect of rEhAPSK and rHsAPSK on APSK activity. (D) Validation of screening system for APSK inhibitors.

A rapid, reproducible *in vitro* assay is critical for screening chemical libraries for compounds that inhibit target enzyme activity. Accordingly, we adjusted a coupling assay to measure APSK activity in a 96-well plate format, in which activity was stoichiometrically determined by monitoring NADH decrease (see Fig 2A; [32]). As well as rEhAPSK, rHsAPSK was prepared and used to generate comparative control data. rEhAPSK was prepared as an affinity-purified His-tagged recombinant whole protein that consists of nonfunctional AS-like and catalytic APSK domains (Fig 1A) (AmoebaDB ID, EHI_179080). Purified rEhAPSK (Fig 2B) showed a specific activity of 4000–6600 nmol/min/mg protein.

rHsAPSK was prepared as an affinity purified His-tagged recombinant APSK domain of HsPAPSS1 for the following reasons. HsPAPSS1 is one of two isoforms present in human, and both HsPAPSS1 and HsPAPSS2 consist of AS and APSK domains, but unlike EhAPSK and AaPAPSS the APSK domain precedes the AS domain in both proteins (Fig 1A) (Genbank Accession No, NP_005434 and NP_004661, respectively; [38–40]). APSK domains of HsPAPSS1 (Ala^25^—Pro^227^) and -2 (Ser^15^—Pro^217^), both of which were individually synthesized as His-tagged recombinant proteins and purified (Fig 2B), showed specific activities of 245–479 and 0–40.9 nmol/min/mg protein, respectively. Furthermore, full-length HsPAPSS1, which was similarly prepared (Fig 2B), did not show detectable activity. Purified rHsAPSK showed ~10–20-fold lower specific activity than rEhAPSK.

Both rEhAPSK and rHsAPSK gave a linear NADH decrease, and slopes became steeper as the amount of enzyme in the coupling solution increased (Fig 2C). The dose-dependent increase of rEhAPSK activity plateaued at 1 μg protein/reaction mixture, and the activity decreased at 5 μg protein/reaction mixture (Fig 2C), indicating the presence of toxic substances in purified rEhAPSK fractions. In contrast, the dose-dependent increase of rHsAPSK activity did not reach a plateau with the same concentration range that was used for rEhAPSK (Fig 2C), which is consistent with ~10–20-fold lower specific activity of rHsAPSK compared with that of rEhAPSK. All three controls, a mock (prepared from *E*. *coli* expressing EhSF2), a solvent (elution buffer used in affinity chromatography), and a blank [50 mM Tris/HCl buffer (pH 8.0)], gave almost constant levels of NADH within the time period monitored (Fig 2D). These results confirm that the previously reported coupling assay to measure APSK activity [32] can be used in a 96-well plate format. The amount of rEhAPSK and rHsAPSK in the reaction mixture was set to give activity in the range of 1.5–3.0 nmol/min; for instance, 0.5 μg rEhAPSK and 5 μg rHsAPSK.

Using the 96-well plate-based APSK activity assay, denatured rEhAPSK and rHsAPSK were independently assayed. Both samples gave almost constant levels of NADH, the profiles of which were comparable to those of the above controls (Fig 2D), simulating complete inhibition of APSK activity. We then screened the same 400 compounds (the Pathogen Box of MMV) that were used in the *in silico* molecular docking analysis. Each compound was assayed in this system at the final concentration of 10 μM. DMSO, which was used to prepare each compound stock, was added as a solvent control at 1% (vol/vol), the final DMSO concentration in all compounds assayed. The inhibitory effect of different compounds on APSK activity was evaluated by the residual activity expressed as the percentage of the activity in each sample relative to that in a DMSO control (set as 100%).

**Fig 3:**
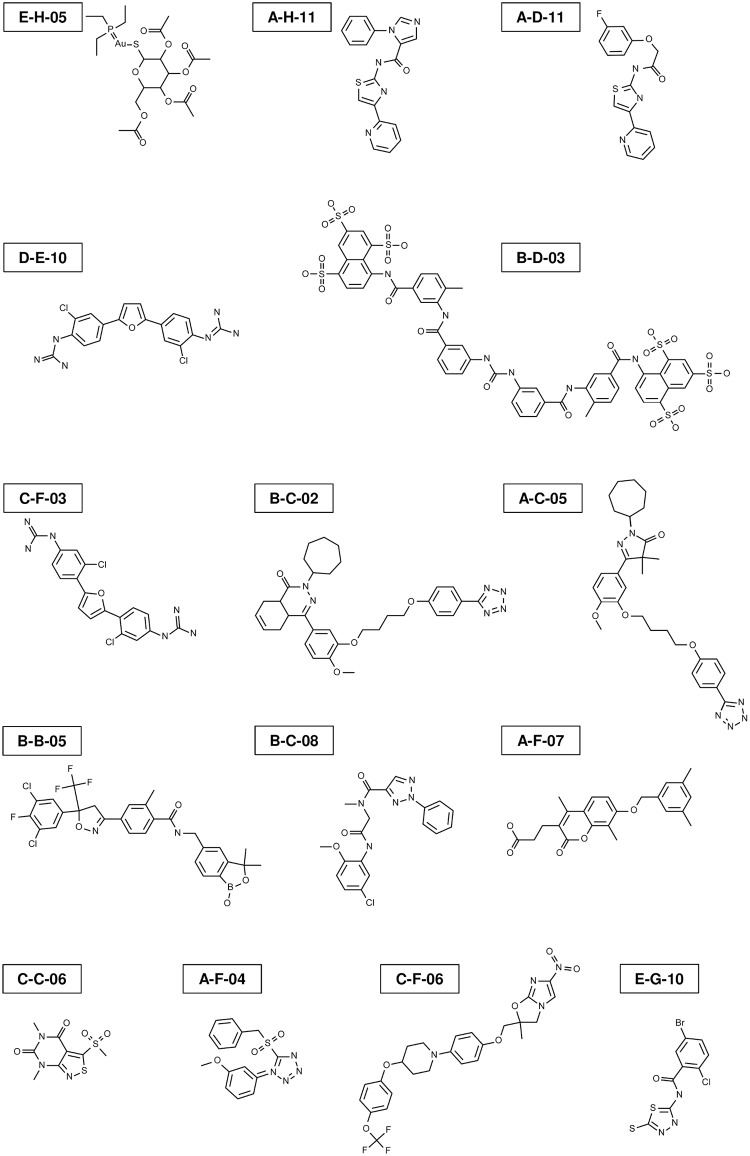
Chemical structures of 15 compounds that reproducibly inhibited rEhAPSK activity.

Fifteen compounds reproducibly inhibited rEhAPSK activity: A-C-05, A-D-11, A-F-04, A-F-07, A-H-11, B-B-05, B-C-02, B-C-08, B-D-03, C-C-06, C-F-03, C-F-06, D-E-10, E-G-10, and E-H-05 (S4 Fig, see Fig 3 for their structures). None of the compounds in the Pathogen Box, including these 15, inhibited rHsAPSK activity (S4 Fig). This rules out any indirect effect of the 15 compounds on the reduction of EhAPSK activity via inhibition of enzymes involved in the coupling reactions of the assay, *i*.*e*., NP1, PK, and LDH (see Fig 2A). Furthermore, these results indicate that these 15 compounds specifically inhibit EhAPSK, and not HsAPSK.

IC_50_ values of the above 15 compounds for rEhAPSK activity were determined, and 12 had IC_50_ values <10 μM: A-C-05, A-D-11, A-F-04, A-H-11, B-B-05, B-C-02, B-C-08, B-D-03, C-C-06, C-F-03, D-E-10, and E-H-05 (Table 2, see Fig 3 for their structures).

**Table 2:** Comparison of results from *in vitro* activity assay and *in silico* docking analysis. Ranking (*in vitro*) was based on IC_50_ values, which were determined by more than three independent experiments and expressed as average ± SD. Ranking (*in silico*) is from S2 Table. A-D-11, A-H-11, and E-H-05 used for IC_50_ determination were from commercial sources whereas the other 12 compounds were from the Pathogen Box. A, EhAPSK structure-A; B, EhAPSK structure-B; and C, EhAPSK structure-C. PDB IDs shown inside parentheses indicate the template tertiary structures used for generating the homology modeling EhAPSK structures.

Pathogen Box		Ranking of potency as EhAPSK inhibitor	APSK activity IC_50_ (μM)	A (3UIE)		B (4FXP)		C (2GKS)	
Compound	Trivial name			Ranking (*in silico*)	Binding energy	Ranking (*in silico*)	Binding energy	Ranking (*in silico*)	Binding energy
D-E-10		1	0.470 ± 0.0499	**13**	-9.5	130	-8.98	30	-8.95
B-D-03	suramin	2	0.520 ± 0.102	**2**	-11.1	69	-9.57	**2**	-10.6
E-H-05	auranofin	3	1.38 ± 0.478	391	-5.13	392	-6.06	329	-6.62
C-F-03		4	2.03 ± 0.205	49	-8.87	90	-9.28	59	-8.45
B-C-02		5	4.27 ± 0.946	**3**	-10.3	**1**	-12	**4**	-10.5
A-C-05		6	4.73 ± 1.16	**1**	-11.4	**4**	-11.4	**13**	-9.33
B-B-05		7	5.73 ± 1.36	56	-8.82	27	-10.3	**10**	-9.74
A-D-11		8	6.30 ± 1.53	300	-6.87	308	-7.7	281	-6.95
B-C-08		9	6.57 ± 1.25	**14**	-9.43	42	-9.94	263	-7.06
A-H-11		10	6.75 ± 1.35	105	-8.21	144	-8.88	163	-7.67
A-F-07		11	7.67 ± 1.25	58	-8.8	**6**	-11	**11**	-9.44
C-C-06		12	8.67 ± 2.62	356	-6.36	371	-6.74	380	-5.84
A-F-04		13	>10	137	-7.94	239	-8.29	101	-8.08
C-F-06	delamanid	13	>10	**4**	-10.3	88	-9.31	**8**	-9.87
E-G-10		13	>10	187	-7.59	322	-7.59	319	-6.71

### Effectiveness of a homology modeling EhAPSK-based *in silico* molecular docking analysis for screening potential leads from a chemical library for the development of new drugs against amoebiasis

We then compared the results for the above 15 compounds (Table 2) obtained from the *in silico* molecular docking analysis (see S2 Table) and the *in vitro* enzyme assay. Among these 15 compounds, six were ranked in the top 20 predicted by the docking analysis using EhAPSK structure-A and -C, which were generated from 3UIE and 2GKS templates, respectively, whereas only three compounds ranked in the top 20 predicted by docking analysis using EhAPSK structure-B, generated from the 4FXP template. Additionally, A-C-05, which had the highest predicted binding affinity by the EhAPSK structure-A-based docking analysis, was included in the above 15 compounds, while D-C-11, which had the highest predicted affinity by the EhAPSK structure-C-based docking analysis, was not (Table 2). These results indicate that *in silico* molecular docking analysis using EhAPSK structure-A, a homology modeling structure of EhAPSK generated from the 3UIE template, is effective for selecting potential leads from a chemical library for the development of new drugs against amoebiasis.

### Compounds targeting EhAPSK can be leads for the development of new drugs against amoebiasis

In our recent study, among the above 15 compounds that reproducibly inhibited EhAPSK activity at 10 μM, A-D-11, A-H-11, and E-H-05 were shown to exert negative effects on processes essential for maintenance of the *Entamoeba* life cycle, *i*.*e*., trophozoite proliferation and cyst formation, at the same concentration, while the other twelve (A-C-05, A-F-04, A-F-07, B-B-05, B-C-02, B-C-08, B-D-03, C-C-06, C-F-03, C-F-06, and D-E-10, E-G-10) were not probably because of APSK compartmentalization in *Entamoeba* mitosomes [10, 11, 18].

**Fig 4:**
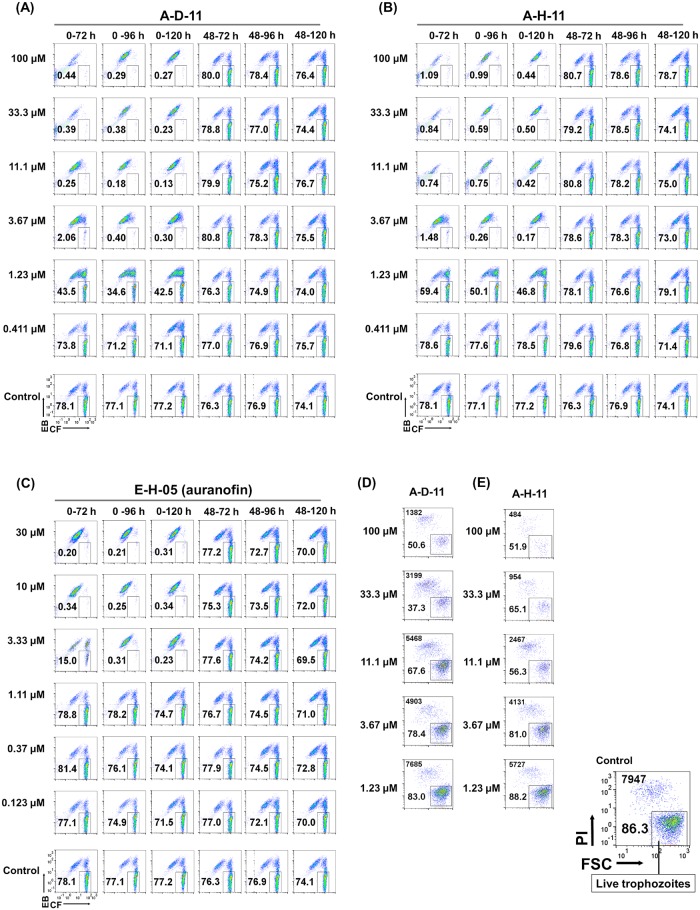
Effects of A-D-11, A-E-11, and E-H-05 (auranofin) on *E*. *invadens* cyst formation and of A-D-11 and A-E-11 on *E*. *histolytica* trophozoite proliferation. (A-C) Encystation assay. Encystation-inducing culture was treated with either A-D-11, A-E-11, or E-H-05 (auranofin), immediately after induction (0−72, 0−96, or 0−120 h). Encystation-inducing culture was treated with either A-D-11, A-E-11 or E-H-05 (auranofin) from 48 h after induction (48–72, -96, or -120 h). Flow cytometry results obtained at 72, 96, or 120 h after induction are shown. EB, Evans blue; CF, calcofluor. (D, E) Trophozoite proliferation assay. The total cell number counted is indicated in the upper left corner. PI, propidium iodide; FSC, forward scatter. Representative data are shown from three independent experiments. Results for E-H-05 treatments (0−72 and 48−72 h), which we previously published [18], were confirmed in this study and are shown for ease of comparison.

IC_50_ values of A-D-11, A-H-11, and E-H-05 for EhAPSK activity, *E*. *histolytica* trophozoite proliferation, and *E*. *invadens* cyst formation were determined using commercially available compounds to avoid a shortage of materials (Tables 2 and 3). Determination of IC_50_ values for *E*. *histolytica* trophozoite proliferation and *E*. *invadens* cyst formation was done by flow cytometry as described [18](Fig 4A (0–72 h), Fig 4B (0–72 h), Fig 4D and 4E for A-D-11 and A-H-11; [18] for E-H-05; Table 3).

**Table 3:** Summary for IC_50_ values of A-D-11, A-H-11, and E-H-05 (auranofin) for processes crucial in *Entamoeba* life cycle and for human cell. IC_50_ values for trophozoite proliferation, cyst formation, and human cell were determined by three independent experiments and expressed as average ± SD. IC_50_ values of E-H-05 for these processes were from [18].

	Trophozoite proliferation	Cyst formation	Cytotoxicity (HFF)
A-D-11	**7.50 ± 2.29 μM**	**2.27 ± 0.252 μM**	**>100 μM**
A-H-11	**4.00 ± 1.05 μM**	**2.35 ± 0.132 μM**	**>100 μM**
E-H-05 (auranofin)	**0.65 ± 0.18 μM**	**1.73 ± 0.70 μM**	**4.00 ± 1.40 μM**

To determine the effect of A-D-11, A-H-11, and E-H-05 on mature cysts, each compound was added to encystation-inducing cultures at 48 h post-induction and cells were then analyzed at 72, 96, and 120 h by flow cytometry as described [18]. E-H-05 did not affected the number of cyst formed at 72 h as previously described (Fig 4C (48–72 h); [18]). Similarly, neither A-D-11 nor A-H-11 affected the number of cysts formed at 72 h (Fig 4A and 4B (48–72 h)). Furthermore, prolonging the duration of compound exposure to 96 and 120 h did not decrease cyst formation efficiency (Fig 4A–4C (48−96 and 48−120 h)), indicating that none of the three compounds had a direct effect on differentiated *Entamoeba* cells that possess similar characteristics to mature cysts. The observed tolerance against these three compounds can be attributed to the presence of a chitin wall that blocks the penetration of the substances into the cell. Taken together, these results indicate that *Entamoeba* cells that are susceptible against the three compounds (A-D-11, A-H-11, and E-H-05) include trophozoites and cells that undergo differentiation from trophozoites but that do not yet show characteristics of mature cysts.

**Fig 5:**
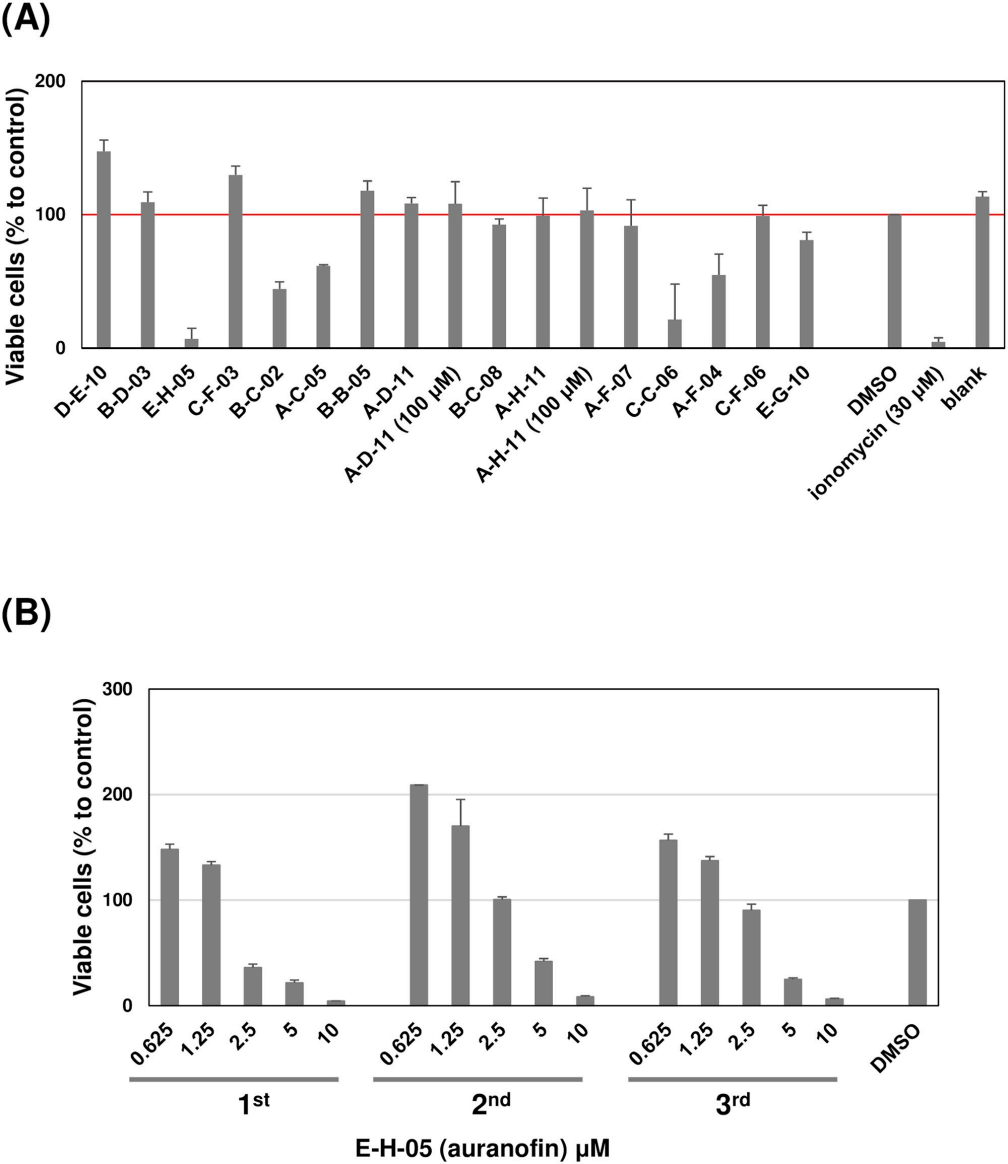
Cytotoxicity of 15 compounds that inhibited rEhAPSK activity against HFF cells. (A) Compound treatments at 10 and 100 μM. (B) Dose-dependent effect of E-H-05 on number of viable HFF cells. Data are expressed relative to that from a DMSO-treated control (set as 100%). Data shown are the mean with error bar. Error bars indicate deviation from the mean, obtained from duplicates from three independent experiments.

We then analyzed cytotoxicity against the host using a WST-1 assay with human HFF cells. Treatment with 30 μM ionomycin, a known ionophore, almost completely abolished cell viability, while 1% DMSO treatment did not show any negative effect, compared with a blank sample (Fig 5), validating the cytotoxicity assay system. In this assay, each of the 15 compounds that inhibited rEhAPSK activity was added at 10 μM. Cytotoxicity was evaluated from number of viable cells relative to that in cells treated with 1% DMSO as a negative control (set as 100%). E-H-05 treatment significantly reduced number of viable cells to <10%, and A-C-05, A-F-04, B-C-02, and C-C-06 treatments moderately reduced numbers of viable cells to ~20 − ~60%, while the other 10 compounds (A-D-11, A-F-07, A-H-11, B-B-05, B-C-08, B-D-03, C-F-03, C-F-06, D-E-10, and E-G-10) did not affect numbers of viable cells (Fig 5). Importantly, A-D-11 and A-H-11, which exerted significant negative effects on *Entamoeba* biological processes, did not show cytotoxicity against the human cells, while E-H-05, which exerted negative effects, showed cytotoxicity as previously reported [41] (Figs 4A–4E and 5; Table 3).

To obtain more information on the cytotoxicity of A-D-11, A-H-11, and E-H-05, commercially available compounds were similarly used for the *Entamoeba* bioassays. E-H-05 showed a cytotoxic effect on HFFs, as previously described [41] and gave an IC_50_ value for HFF cell of 4.00 ± 1.40 μM (Table 3). In contrast, A-D-11 and A-H-11 treatments did not influence numbers of viable HFF cells; the same effect was observed at 10 μM and 100 μM (Fig 5; Table 3), indicating almost no cytotoxic activity of A-D-11 and A-H-11 against *E*. *histolytica* human host cells. Considering the *Entamoeba* bioassay data, these results indicate that the cytotoxic actions of A-D-11 and A-H-11 are highly selective towards *Entamoeba* cells (Table 3). Hence, both A-D-11 and A-H-11 are promising leads for the development of new drugs against amoebiasis.

### EhAPSK is a rational target for amoebiasis therapy

**Fig 6:**
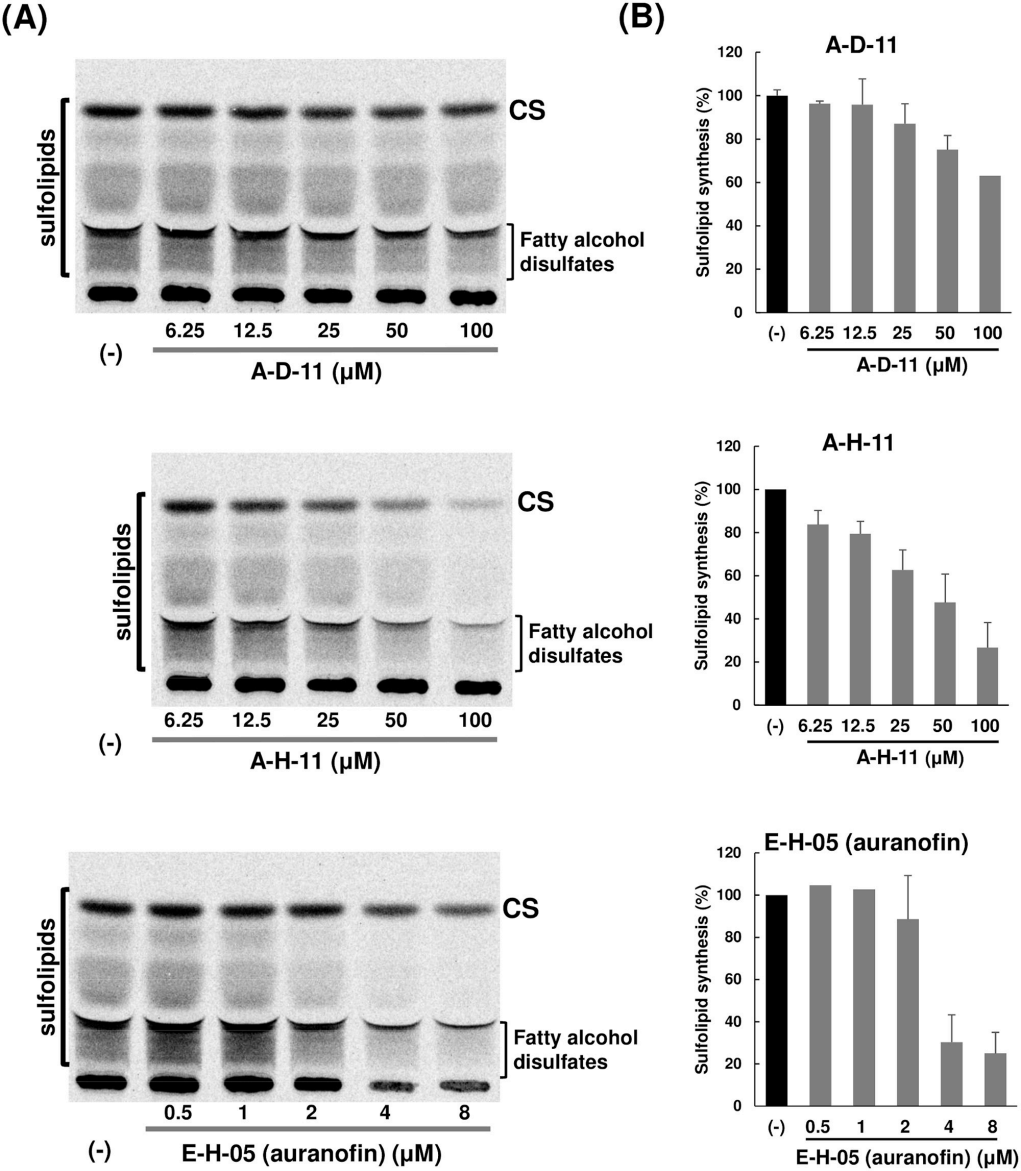
Sulfolipid synthesis in *E*. *histolytica* treated for 6 h with A-D-11, A-H-11, or E-H-05 (auranofin). (A) Autoradiography of HPTLC of methanol extracts of [^35^S]-labeled trophozoites cultivated in the presence of different concentrations of each compound. The autoradiographic images shown are representative of the results from three independent experiments. (B) Quantitation of sulfolipids synthesized in trophozoites cultivated in the presence of different concentrations of each compound. The data were calculated from the autoradiography image and are expressed relative to that in a solvent-treated control (set as 100%) after normalizing to total synthesized sulfolipids and total cell protein. Data shown are the mean with error bar (SD from the mean) from three independent experiments. Samples for A-D-11 and A-H-11 treatments were originally run in a single TLC plate, which included a lane for a control sample, and the obtained image was split into two parts for ease of comparison; therefore, the single control lane was used to calculate the relative values for both A-D-11 and A-H-11.

Subsequently, we investigated the causal link between EhAPSK inhibition and the halting of not only *E*. *histolytica* trophozoite proliferation but also *E*. *invadens* cyst formation, which are essential for the maintenance of the *Entamoeba* life cycle. As key molecules mediating this linkage, we focused on sulfolipids, terminal products of *Entamoeba* sulfur metabolism involving EhAPSK: fatty alcohol disulfates involved in *E*. *histolytica* trophozoite proliferation and cholesteryl sulfate involved in *Entamoeba* encystation [8, 9]. *E*. *histolytica* trophozoites were metabolically labeled by [^35^S]-labeled sulfate in the presence of each of the three compounds (A-D-11, A-H-11, and E-H-05). All these compounds dose-dependently reduced the amount of sulfolipids synthesized (Fig 6). The concentration ranges tested almost overlapped with those produced trophozoite morphology similar to that in DMSO control-treated cells (6.25–100 and 0.5–4 μM, respectively, for A-D-11 and A-H-11 and for E-H-05) (S5 Fig). These results indicate that A-D-11, A-H-11, and E-H-05 all inhibited APSK activity in live *E*. *histolytica* cells, and that E-H-05 showed higher potency than A-H-11 and A-D-11 for impairment of sulfolipid synthesis, which correlated with their IC_50_ values for trophozoite proliferation and EhAPSK activity (see Fig 4D and 4E and Table 3). A previous study suggests that auranofin, which is E-H-05 in the Pathogen Box, exerts a cytotoxic effect on trophozoites by targeting thioredoxin (Trx) reductase (TrxR) in *E*. *histolytica* [19]; therefore, these results also indicate that the mode of action of E-H-05 differs from those of A-D-11 and A-H-11; E-H-05 targets two enzymes, EhAPSK and *E*. *histolytica* TrxR (EhTrxR). Taken together, A-D-11, A-H-11, and E-H-05 halt biological processes essential to the *Entamoeba* life cycle by targeting EhAPSK, which impairs the synthesis of sulfolipids, such as fatty alcohol disulfates and cholesteryl sulfate. The *Entamoeba* processes halted by the three compounds, *i*.*e*., trophozoite proliferation and cyst formation, are closely associated with the clinical manifestation and pathogenesis of amoebiasis, and with disease transmission, respectively. Hence, EhAPSK is a rational target for amoebiasis therapy.

## Discussion

Generation of both appropriate targets and leads is essential for the development of new drugs. To provide desirable leads, large scale screening is a prerequisite; therefore, *in vitro*-based high throughput systems or *in silico* molecular docking analysis are widely adopted. Here, we performed both types of analysis, *i*.*e*., an *in vitro* APSK activity assay and EhAPSK-based *in silico* molecular docking analysis, using a pilot chemical library consisting of 400 compounds. The *in vitro* assay enabled us to identify 15 compounds as EhAPSK inhibitors. These 15 compounds are reported to be effective for various infectious diseases. The causative agents of these diseases include *Mycobacterium tuberculosis*, *Burkholderia pseudomallei*, *Staphylococcus aureus*, *Cryptococcus neoformans*, *Candida albicans*, *Toxoplasma gondii*, *Plasmodium falciparum*, *Giardia intestinalis*, *Trypanosoma brucei*, *Trypanosoma cruzi*, and *Onchocerca volvulus* (S4 Table) [42–49]. It is intriguing to speculate whether the compounds target APSK in these pathogens.

EhAPSK-based *in silico* molecular docking analysis together with the above *in vitro* APSK activity assay showed that *in silico* molecular docking using a homology modeling structure of EhAPSK generated from a 3UIE template is effective for selecting EhAPSK inhibitors, which are potential leads for the development of new drugs against amoebiasis. Effectiveness will be underscored when a huge number of compounds, *e*.*g*., more than a million, needs to be screened because it is not practical to measure the inhibitory activity of such a large number of compounds by the present *in vitro* assay; therefore, our docking analysis procedure has an important role as a primary screening system. However, it is critical to set a threshold for *in silico* primary screening that does not overlook candidate compounds because, as shown in the present study, binding energies calculated by *in silico* molecular docking analysis do not always correlate with IC_50_ values determined by the *in vitro* activity assay. This may be because the IC_50_ value, which is affected by various kinetic constants, *i*.*e*., K_m_, K_i_, and V_max_, is not an appropriate parameter. Instead, an experimentally determined binding constant, such as K_d_, may correlate more closely to the binding energy calculated by *in silico* molecular docking analysis. Another plausible explanation for this reduced correlation is a structural difference between the homology modeling of EhAPSK and rEhAPSK. A limitation of the program used in the *in silico* analysis also risks overlooking candidate compounds that cannot simulate boron-containing and Au-bound molecules *per se*, which may wrongly give a higher energy than threshold; for example, E-H-05, which contains Au, ranked in the bottom quartile for binding energy, but showed significant inhibitory activity against rEhAPSK.

To characterize the effect(s) of screened compounds on biological processes in a living organism, different types of assays are needed. In the present study, the effects on *Entamoeba* trophozoite proliferation and cyst formation were determined to characterize the fifteen compounds that significantly inhibited EhAPSK activity *in vitro*. This strategy provided evidence that three compounds (A-D-11, A-H-11, and E-H-05) halted both trophozoite proliferation and cyst formation, in addition to inhibiting EhAPSK. Furthermore, these three compounds also impaired sulfolipid synthesis, including for fatty alcohol disulfates and cholesteryl sulfate, indicating that their target in *Entamoeba* cells is APSK, and that the three compounds are leads for the development of new drugs against amoebiasis. Notably, these results are consistent with gene silencing of EhAPSK, which retarded the proliferation of *E*. *histolytica* trophozoites [11], and that indirect inhibition of APSK activity in *E*. *invadens*, mediated by lowering the level of a substrate for APSK by treating an *in vitro E*. *invadens* culture with an AS inhibitor, resulted in reducing the number of cysts formed [8].

Auranofin, which is E-H-05 in the Pathogen Box, is one of the three lead compounds. Auranofin was previously suggested to exert a cytotoxic effect on *E*. *histolytica* trophozoites by enhancing reactive oxygen-mediated cell killing via inhibition of EhTrxR [19]. Auranofin possesses broad parasiticidal and bactericidal activities, and its potency is mediated by destroying intracellular redox homeostasis via targeting thiol redox enzymes such as TrxR, thioredoxin-glutathione reductase, and trypanothione reductase [50–58]. X-ray structure analysis shows that auranofin binds to cysteine thiol groups in the catalytic C(X)_4_C motif of *S*. *mansoni* thioredoxin-glutathione reductase [50] and to the trypanothione binding site of *L*. *infantum* trypanothione reductase [51]. However, x-ray structure analysis was not available for either the binding of auranofin to cysteine thiol groups in the catalytic C(X)_2_C motif or to a substrate- (Trx) binding site of EhTrxR. Nevertheless, the inhibition of EhTrxR by auranofin occurred during turnover of Trx in an *in vitro* assay using purified recombinant enzyme [59]. These findings indicate that the molecular mechanism of auranofin action on EhTrxR may differ from that on its targets in other organisms. The present results, including the IC_50_ value of auranofin for rEhAPSK, which is similar to that of recombinant EhTrxR (1.38 ± 0.478 vs 0.4 μM, respectively [19, 59]), suggest that in *E*. *histolytica*, auranofin targets two distinct enzymes: EhAPSK and EhTrxR.

Compartmentalization of *Entamoeba* APSK into mitosomes, which are derived from canonical mitochondria [10, 11], may explain the apparently inconsistent results of the 12 compounds that at 10 μM inhibited EhAPSK in the *in vitro* activity assay system but did not exert negative effects on trophozoite proliferation or cyst formation (this study; [18]); these compounds may not be able to reach their targets in *Entamoeba* cells because of three phospholipid bilayers, *i*.*e*., the cell membrane, and mitosomal inner and outer membranes. Alternatively, being metabolized, exported, or bound to nonspecific factors before reaching their target may render these compounds unable to inhibit APSK in *Entamoeba* cells. These potential mechanisms may also generate different inhibitory profiles for the three compounds (A-D-11, A-H-11, and E-H-05) on EhAPSK activity and on other biological processes because each compound has different chemical properties that show distinct activities against cellular features. The dual target hypothesis is, however, a more plausible explanation for the different profiles of E-H-05 and the other two compounds. Taken together, EhAPSK is a rationale target for amoebiasis therapy. However, several issues should be considered for compounds from an initial screen to be considered as leads, such as chemical properties in relation to chitin wall and membrane permeability, and stability in *E*. *histolytica*.

Selectivity is among the most important issues in providing suitable leads for drug development. The three lead compounds identified in this study (A-D-11, A-H-11, and E-H-05) show selectivity because none showed any inhibitory activity against HsAPSK in the *in vitro* assay system. Furthermore, A-D-11 and A-H-11 showed no cytotoxic activity against HFF cells. These results, however, cannot be necessary interpreted as high selectivity toward EhAPSK because a structural difference cannot be ruled out between native HsPAPSS1, a bi-functional APSK-AS fusion protein, and rHsAPSK, the APSK domain of full-length HsPAPSS1 used in the assay. The above three compounds bind to the crystal structure of HsPAPSS1 (UniProtKB ID, O43252; PDB ID, 1XNJ) [60] at affinities comparable to that of EhAPSK in the *in silico* molecular docking analysis, but did not show any inhibitory activities in the *in vitro* APSK activity assay (S3 Table). Reliance on a single method to screen a large chemical library, therefore, possesses a risk of false-positive and -negative results.

In conclusion, a combination screen of EhAPSK-based *in silico* molecular docking analysis combined with an *in vitro* activity assay identified compounds that potentially affect the *Entamoeba* life cycle, *i*.*e*., trophozoite proliferation and cyst formation, and sulfolipid synthesis. Further characterization of these compounds can provide desirable leads for the development of new anti-amoebic and amoebiasis transmission-blocking drugs. In addition, the present strategy, involving applied and basic *Entamoeba* biology can provide important and intriguing issues to be addressed. The present strategy can also be applied to develop a system for identifying specific APSK inhibitors. Materials provided by both the present and future studies will help advance the understanding of sulfur metabolism not only in *Entamoeba* but also in other organisms, a fundamental topic of general biochemistry and physiology.

## Supporting information

S1 Table List of primers. (PDF)

S2 Table Binding free energy of all compounds in the MMV Pathogen Box toward computer modeled structures of EhAPSK. A, EhAPSK structure-A; B, EhAPSK structure-B; and C, EhAPSK structure-C. PDB IDs shown inside parentheses indicate the tertiary structures used as templates for generating each of the three EhAPSK structures.
(PDF)

S3 Table Comparison of EhAPSK-based rankings among *in vitro* and *in silico* data and HsPAPPS-1-based rankings. Ranking of *in vitro* data for EhAPSK and HsAPSK is based on inhibition levels against rEhAPSK (from Table 2) and rHsAPSK (from S4 Fig) activities, respectively. Ranking of *in silico* data are based on the binding energies determined by computer simulated docking analysis between either EhAPSK structure-A or the tertiary structure of HsPAPSS1 (PDB ID, 1XNJ) and each compound in the Pathogen Box. NS, not significantly inhibited.
(PDF)

S4 Table Information on the 15 compounds that inhibit rEhAPSK activity. (PDF)

S1 Fig Multiple sequence alignment of the APSK domain from EhAPSK and homologs from various organisms. The UniProtKB ID number for each protein is indicated inside parentheses. The amino acid residues conserved in all proteins are indicated by *.
(TIF)

S2 Fig Different spatial positions of Arg^420^ and Lys^421^ side-chains in the cavities of homology modeled EhAPSK structures. Close up of the spatial positions of side-chains in the cavity of homology-modeled EhAPSK structure-A (blue) and -B (red).
(TIF)

S3 Fig Structural similarity of two homology-modeled EhAPSKs differently accommodating compound A-D-11. Binding pattern of A-D-11 to EhAPSK-C, which is structurally different from both EhAPSK-A and -B, is also shown for ease of comparison. A-D-11 positioned in each cavity of the homology modeled EhAPSKs is highlighted in red.
(TIF)

S4 Fig Screening 400 compounds with diverse scaffolds from the MMV Pathogen Box by the *in vitro* APSK activity assay. (A-E) The effect(s) on activities of rEhAPSK (upper) and rHsAPSK (lower). Data are expressed as the residual activity expressed as the percentage of the activity in each sample relative to that in DMSO control (set as 100%). Data shown are the mean with error bar (SD from the mean) from three independent experiments. Red bars and arrows indicate compounds that reproducibly inhibited rEhAPSK activity. DMSO and blank controls were included. Five 96-well plates (plate A-E) were used, into which 400 compounds together with DMSO and blank controls were equally dispensed (80 wells per plate).
(PDF)

S5 Fig Morphology of cells treated with each compound. Phase contrast images of cells are shown that were treated for 6 h with A-D-11, A-H-11, or E-H-05 (auranofin) at the indicated concentrations. Bar indicates 50 μm. Representative images from three independent experiments are shown.
(TIF)
