# Supplementary material for: Characterization of Entamoeba histolytica adenosine 5′-phosphosulfate (APS) kinase; validation as a target and provision of leads for the development of new drugs against amoebiasis
Source: PLoS Negl Trop Dis. 2019 Aug 19;13(8):e0007633. doi: 10.1371/journal.pntd.0007633 (PMC6715247; doi:10.1371/journal.pntd.0007633)
Supplement: S1 Table — (PDF) [file pntd.0007633.s001.pdf]

Target gene or domain	Name	Sequence	Direction	Mention Underline
EhAPSK	Pc-EhAPSK-F	5'-ATCCTCGAGATGGCTACTGCTAAGATTGCTG-3'	sense	XhoI restriction site
	Pc-EhAPSK-R	5'-ATCCTGCAGTCATTTAATGTATTGCTTCTTTGTG-3'	antisense	PstI restriction site
APSK-domain of HsPAPSS1	Pc-HsAPSK1-F	5'-ATACTCGAGGCAACCAATGTCACCTACCAAGC-3'	sense	XhoI restriction site
	Pc-HsAPSK1-R	5'-ATACTGCAGCTAAGGTACAATATCCCGTTCCTGTA-3'	antisense	PstI restriction site
APSK-domain of HsPAPSS2	Pc-HsAPSK2-F	5'-ATACTCGAGTCCACCAATGTAGTCTATCAGGC-3'	sense	XhoI restriction site
	Pc-HsAPSK2-R	5'-ATACTGCAGCTAGGGTACAATGTTCTGCTCTTGCA-3'	antisense	PstI restriction site
HsPAPSS1	Pc-HsPAPSS1-F	5'-ATACATATGGAGATCCCCGGGAG-3'	sense	NdeI restriction site
	Pc-HsPAPSS1-R	5'-ATAGTCGACCTAAGCTTTCTCCAAGGATTTG-3'	antisense	SalI restriction site
EhSF2	Pc-EhSF2-F	5'-ATCGGATCCATGGCTTCTCAATTTACAATAGAAACAAACA-3'	sense	BamHI restriction site
	Pc-EhSF2-R	5'-ATCGTCGACTTAGTTCTTCGGTTCAATAATAGAATAACG-3'	antisense	SalI restriction site
