# Supplementary material for: Characterization of Entamoeba histolytica adenosine 5′-phosphosulfate (APS) kinase; validation as a target and provision of leads for the development of new drugs against amoebiasis
Source: PLoS Negl Trop Dis. 2019 Aug 19;13(8):e0007633. doi: 10.1371/journal.pntd.0007633 (PMC6715247; doi:10.1371/journal.pntd.0007633)
Supplement: S2 Table — A, EhAPSK structure-A; B, EhAPSK structure-B; and C, EhAPSK structure-C. PDB IDs shown inside parentheses indicate the tertiary structures used as templates for generating each of the three EhAPSK structures. (PDF) [file pntd.0007633.s002.pdf]

Ranking of affinity	A (3UIE) Compound	A (3UIE) Binding energy	B (4FXP) Compound	B (4FXP) Binding energy	C (2GSK) Compound	C (2GSK) Binding energy
**substrate**	APS	-9.74	APS	-11.98	APS	-9.38
**1**	Plate A C05	-11.36	Plate B C02	-11.98	Plate D C11	-10.94
**2**	Plate B D03	-11.11	Plate E B11	-11.91	Plate B D03	-10.64
**3**	Plate B C02	-10.34	Plate B A02	-11.53	Plate A F02	-10.53
**4**	Plate C F06	-10.28	Plate A C05	-11.41	Plate B C02	-10.51
**5**	Plate A E11	-10.23	Plate B A11	-11.04	Plate D F08	-10.06
**6**	Plate D G09	-9.78	Plate A F07	-11.03	Plate E B11	-10.01
**7**	Plate D B07	-9.77	Plate B F10	-11.02	Plate B A11	-9.95
**8**	Plate A F02	-9.7	Plate D C11	-10.86	Plate C F06	-9.87
**9**	Plate E B11	-9.66	Plate E D06	-10.83	Plate B A09	-9.81
**10**	Plate C B10	-9.6	Plate A D04	-10.79	Plate B B05	-9.74
**11**	Plate D C11	-9.57	Plate C D10	-10.79	Plate A F07	-9.44
**12**	Plate A H03	-9.52	Plate A G02	-10.78	Plate A G02	-9.37
**13**	Plate D E10	-9.5	Plate D F02	-10.7	Plate A C05	-9.33
**14**	Plate B C08	-9.43	Plate A F02	-10.66	Plate B A08	-9.27
**15**	Plate C B11	-9.39	Plate D A08	-10.59	Plate D C06	-9.27
**16**	Plate D E07	-9.39	Plate A B08	-10.58	Plate D C07	-9.25
**17**	Plate D E05	-9.38	Plate D F09	-10.58	Plate C F09	-9.23
**18**	Plate B A11	-9.31	Plate B D08	-10.55	Plate B B06	-9.15
**19**	Plate D F08	-9.31	Plate A E11	-10.53	Plate E B05	-9.09
**20**	Plate E D10	-9.29	Plate B F11	-10.44	Plate D F09	-9.08
**21**	Plate C E06	-9.28	Plate A H03	-10.32	Plate E A07	-9.08
**22**	Plate A G02	-9.27	Plate D C05	-10.32	Plate D F02	-9.07
**23**	Plate D D09	-9.23	Plate D D09	-10.31	Plate D D10	-9.06
**24**	Plate D F09	-9.23	Plate C B09	-10.3	Plate A E11	-9.04
**25**	Plate C C08	-9.21	Plate D D10	-10.29	Plate B D08	-9.03
**26**	Plate B B09	-9.2	Plate E G09	-10.28	Plate D H02	-9
**27**	Plate C E02	-9.2	Plate B B05	-10.27	Plate B B11	-8.99
**28**	Plate A B09	-9.18	Plate A F11	-10.24	Plate C B10	-8.98
**29**	Plate A A03	-9.17	Plate C F04	-10.24	Plate C E06	-8.98
**30**	Plate B F10	-9.17	Plate D B10	-10.21	Plate D E10	-8.95
**31**	Plate E F04	-9.15	Plate C C08	-10.19	Plate C D10	-8.94
**32**	Plate A A04	-9.13	Plate D C07	-10.16	Plate D C05	-8.94
**33**	Plate B E07	-9.11	Plate C F09	-10.11	Plate C B11	-8.93
**34**	Plate B E09	-9.08	Plate D B09	-10.09	Plate A H02	-8.88
**35**	Plate A D05	-9.03	Plate D G04	-10.07	Plate A H05	-8.88
**36**	Plate A B06	-9.01	Plate A G07	-10.04	Plate B E07	-8.88
**37**	Plate C E03	-8.99	Plate D B07	-10.04	Plate C C08	-8.86
**38**	Plate E C07	-8.99	Plate E F10	-10.04	Plate A A03	-8.85
**39**	Plate A A11	-8.98	Plate C A08	-10.01	Plate C B09	-8.85
**40**	Plate E F10	-8.97	Plate D F10	-9.97	Plate D B07	-8.84
**41**	Plate B D08	-8.96	Plate D E07	-9.96	Plate C B03	-8.81
**42**	Plate E G04	-8.96	Plate B C08	-9.94	Plate C C10	-8.76
**43**	Plate B B11	-8.95	Plate D C06	-9.94	Plate B F11	-8.73
**44**	Plate B A02	-8.94	Plate E F06	-9.94	Plate E A06	-8.72
**45**	Plate C H04	-8.94	Plate B G03	-9.91	Plate A G07	-8.69
**46**	Plate E C03	-8.94	Plate E C04	-9.85	Plate E C05	-8.66
**47**	Plate E G08	-8.91	Plate B H06	-9.81	Plate B A10	-8.62
**48**	Plate D D10	-8.9	Plate D H10	-9.81	Plate D E07	-8.62
**49**	Plate C F03	-8.87	Plate E E05	-9.81	Plate E G09	-8.62
**50**	Plate E H07	-8.87	Plate A H05	-9.79	Plate B F03	-8.6
**51**	Plate A B08	-8.86	Plate D G10	-9.78	Plate D D09	-8.6
**52**	Plate A E09	-8.85	Plate E F07	-9.77	Plate D H04	-8.6
**53**	Plate B C11	-8.84	Plate B A08	-9.74	Plate B E05	-8.57
**54**	Plate A G07	-8.83	Plate E C08	-9.74	Plate B B09	-8.55
**55**	Plate C D04	-8.83	Plate B E07	-9.73	Plate E D06	-8.53
**56**	Plate B B05	-8.82	Plate C B10	-9.72	Plate C B05	-8.5
**57**	Plate D A08	-8.82	Plate A H02	-9.66	Plate B B08	-8.49
**58**	Plate A F07	-8.8	Plate C E06	-9.66	Plate A A05	-8.48
**59**	Plate A D04	-8.79	Plate D G09	-9.66	Plate C F03	-8.45
**60**	Plate B E11	-8.78	Plate B B09	-9.64	Plate B F09	-8.44
**61**	Plate D B10	-8.78	Plate A E09	-9.62	Plate B B07	-8.43
**62**	Plate C D10	-8.76	Plate B A10	-9.62	Plate A B08	-8.38
**63**	Plate D C06	-8.76	Plate B C06	-9.62	Plate D D08	-8.37
**64**	Plate A F11	-8.74	Plate A H06	-9.61	Plate B H06	-8.36
**65**	Plate C B03	-8.73	Plate B B11	-9.6	Plate C F04	-8.35
**66**	Plate E D08	-8.7	Plate C D09	-9.59	Plate D B09	-8.35
**67**	Plate B F09	-8.69	Plate C C10	-9.58	Plate B H07	-8.31
**68**	Plate B F11	-8.69	Plate E E10	-9.58	Plate D H09	-8.31
**69**	Plate E E10	-8.69	Plate B D03	-9.57	Plate E G02	-8.31
**70**	Plate C F09	-8.67	Plate D E05	-9.57	Plate A F09	-8.29
**71**	Plate E F07	-8.65	Plate A A11	-9.56	Plate C H03	-8.28
**72**	Plate B C04	-8.64	Plate B F09	-9.56	Plate B D10	-8.27
**73**	Plate B H06	-8.64	Plate C G04	-9.56	Plate B H09	-8.27
**74**	Plate D H02	-8.64	Plate D G07	-9.56	Plate D F10	-8.27
**75**	Plate B C07	-8.63	Plate A D05	-9.52	Plate A H03	-8.26
**76**	Plate A H02	-8.62	Plate C B03	-9.48	Plate B F10	-8.26
**77**	Plate C E08	-8.62	Plate D A11	-9.48	Plate D B04	-8.26
**78**	Plate D F02	-8.61	Plate C E02	-9.47	Plate D E05	-8.25
**79**	Plate A F03	-8.59	Plate B C09	-9.46	Plate B G03	-8.24
**80**	Plate B E02	-8.56	Plate A F09	-9.44	Plate D C08	-8.24
**81**	Plate B C09	-8.55	Plate E C06	-9.43	Plate D G08	-8.24
**82**	Plate A H05	-8.54	Plate B A09	-9.42	Plate C C09	-8.22
**83**	Plate D C05	-8.51	Plate B C11	-9.4	Plate D D07	-8.21
**84**	Plate E D06	-8.49	Plate C H04	-9.4	Plate A A04	-8.2
**85**	Plate D A05	-8.47	Plate A G05	-9.37	Plate A G11	-8.2
**86**	Plate D G04	-8.46	Plate C B11	-9.37	Plate B C10	-8.2
**87**	Plate D H04	-8.42	Plate E D08	-9.37	Plate A D05	-8.19
**88**	Plate E G09	-8.42	Plate C F06	-9.31	Plate E A05	-8.19
**89**	Plate D F10	-8.41	Plate A B04	-9.3	Plate E C08	-8.19
**90**	Plate B A08	-8.4	Plate C F03	-9.28	Plate A A06	-8.18
**91**	Plate B A09	-8.39	Plate C C03	-9.26	Plate A E09	-8.18
**92**	Plate E E05	-8.37	Plate A A06	-9.25	Plate B F02	-8.18
**93**	Plate C C10	-8.34	Plate C C09	-9.25	Plate B A02	-8.15
**94**	Plate D G07	-8.34	Plate A H08	-9.24	Plate C B02	-8.15
**95**	Plate B B10	-8.32	Plate B C03	-9.24	Plate E A04	-8.15
**96**	Plate A D03	-8.31	Plate B D09	-9.24	Plate E F04	-8.15
**97**	Plate B A10	-8.31	Plate E G02	-9.24	Plate D B08	-8.14
**98**	Plate C A10	-8.31	Plate E F04	-9.23	Plate D H03	-8.1
**99**	Plate D D06	-8.31	Plate C D06	-9.22	Plate C C03	-8.09
**100**	Plate A A05	-8.3	Plate A B09	-9.21	Plate C E09	-8.09

Ranking of affinity	A ((3UIE)			B (4FXP)			C (2GSK)		
	Compound		Binding energy	Compound		Binding energy	Compound		Binding energy
**101**	Plate C	A11	-8.29	Plate D	C08	-9.21	Plate A	F04	-8.08
**102**	Plate D	D07	-8.29	Plate E	C07	-9.21	Plate E	E10	-8.08
**103**	Plate B	B07	-8.26	Plate D	D08	-9.2	Plate C	C07	-8.06
**104**	Plate B	F03	-8.23	Plate D	C04	-9.15	Plate C	G07	-8.05
**105**	Plate A	H11	-8.21	Plate A	E07	-9.14	Plate E	F07	-8.03
**106**	Plate D	B04	-8.21	Plate E	H07	-9.13	Plate D	G09	-8.01
**107**	Plate D	H03	-8.21	Plate B	H10	-9.11	Plate E	A10	-8.01
**108**	Plate E	C06	-8.21	Plate C	F05	-9.11	Plate B	C04	-7.99
**109**	Plate C	H02	-8.2	Plate D	D03	-9.11	Plate B	E09	-7.99
**110**	Plate D	H08	-8.2	Plate E	G08	-9.1	Plate D	D06	-7.99
**111**	Plate C	A05	-8.18	Plate C	D08	-9.09	Plate C	A10	-7.97
**112**	Plate D	G08	-8.18	Plate D	H04	-9.09	Plate E	F10	-7.97
**113**	Plate E	A10	-8.17	Plate B	D10	-9.08	Plate D	H10	-7.96
**114**	Plate A	F10	-8.16	Plate C	H06	-9.08	Plate A	F11	-7.95
**115**	Plate B	G03	-8.16	Plate C	H02	-9.07	Plate B	A07	-7.94
**116**	Plate C	F04	-8.15	Plate A	B06	-9.06	Plate B	B10	-7.94
**117**	Plate A	E07	-8.14	Plate D	A05	-9.06	Plate D	A07	-7.94
**118**	Plate D	C10	-8.14	Plate B	H07	-9.04	Plate C	E02	-7.93
**119**	Plate C	C09	-8.13	Plate C	E08	-9.04	Plate E	H10	-7.93
**120**	Plate D	D08	-8.13	Plate D	D07	-9.04	Plate D	A08	-7.92
**121**	Plate E	B10	-8.13	Plate C	B02	-9.03	Plate D	G10	-7.92
**122**	Plate A	E05	-8.11	Plate B	B07	-9.02	Plate A	A11	-7.91
**123**	Plate C	C05	-8.1	Plate C	C05	-9.02	Plate C	E03	-7.91
**124**	Plate C	H09	-8.1	Plate C	E09	-9.02	Plate C	H04	-7.91
**125**	Plate A	G05	-8.09	Plate D	H03	-9.02	Plate D	F05	-7.91
**126**	Plate B	C03	-8.07	Plate C	B05	-9	Plate C	C05	-7.9
**127**	Plate A	H06	-8.06	Plate B	E02	-8.99	Plate D	G07	-7.9
**128**	Plate C	E11	-8.06	Plate C	F07	-8.99	Plate B	C06	-7.89
**129**	Plate E	C11	-8.05	Plate A	G11	-8.98	Plate D	C03	-7.89
**130**	Plate E	C05	-8	Plate D	E10	-8.98	Plate D	C09	-7.88
**131**	Plate A	C04	-7.98	Plate B	G05	-8.96	Plate B	G05	-7.87
**132**	Plate D	B09	-7.98	Plate C	A10	-8.96	Plate B	C11	-7.86
**133**	Plate D	C09	-7.98	Plate C	H09	-8.96	Plate D	A05	-7.86
**134**	Plate A	G06	-7.97	Plate B	B03	-8.95	Plate D	H06	-7.86
**135**	Plate B	H02	-7.96	Plate A	F10	-8.94	Plate A	F03	-7.85
**136**	Plate C	H03	-7.96	Plate E	C03	-8.94	Plate D	A02	-7.85
**137**	Plate A	D02	-7.95	Plate A	C02	-8.92	Plate E	D03	-7.84
**138**	Plate A	F04	-7.94	Plate B	E09	-8.92	Plate A	D04	-7.83
**139**	Plate C	F02	-7.94	Plate D	A10	-8.92	Plate A	C06	-7.81
**140**	Plate D	B06	-7.93	Plate D	A09	-8.91	Plate C	A11	-7.81
**141**	Plate B	H07	-7.92	Plate C	A11	-8.89	Plate D	B11	-7.81
**142**	Plate D	A09	-7.92	Plate D	C03	-8.89	Plate C	E11	-7.79
**143**	Plate D	C07	-7.92	Plate E	A04	-8.89	Plate E	C07	-7.79
**144**	Plate A	C10	-7.91	Plate A	H11	-8.88	Plate A	A07	-7.77
**145**	Plate B	F06	-7.9	Plate C	E03	-8.88	Plate E	C06	-7.76
**146**	Plate E	E02	-7.9	Plate D	H02	-8.88	Plate A	B06	-7.75
**147**	Plate E	G02	-7.89	Plate E	A09	-8.88	Plate A	H06	-7.75
**148**	Plate B	C10	-7.88	Plate A	E03	-8.87	Plate C	H09	-7.75
**149**	Plate A	C06	-7.84	Plate E	B07	-8.87	Plate D	A04	-7.75
**150**	Plate D	A10	-7.84	Plate A	F06	-8.86	Plate D	D03	-7.74
**151**	Plate D	H10	-7.84	Plate B	A04	-8.86	Plate A	D02	-7.73
**152**	Plate E	F06	-7.84	Plate B	C04	-8.86	Plate D	G04	-7.73
**153**	Plate C	H05	-7.83	Plate B	E11	-8.84	Plate E	F06	-7.73
**154**	Plate D	F05	-7.82	Plate E	D03	-8.84	Plate E	H07	-7.73
**155**	Plate E	D11	-7.82	Plate B	E05	-8.82	Plate A	F06	-7.72
**156**	Plate A	A07	-7.8	Plate D	D06	-8.82	Plate B	D09	-7.72
**157**	Plate C	D08	-7.79	Plate E	H04	-8.82	Plate B	D11	-7.72
**158**	Plate D	C03	-7.79	Plate D	F08	-8.81	Plate B	E11	-7.72
**159**	Plate E	A09	-7.79	Plate E	G04	-8.81	Plate E	B10	-7.71
**160**	Plate A	D08	-7.78	Plate A	D02	-8.79	Plate E	D08	-7.7
**161**	Plate C	D07	-7.78	Plate D	B03	-8.79	Plate B	B04	-7.69
**162**	Plate A	B07	-7.77	Plate B	B10	-8.78	Plate B	E02	-7.68
**163**	Plate A	G04	-7.76	Plate C	E11	-8.78	Plate A	H11	-7.67
**164**	Plate B	B04	-7.76	Plate D	C09	-8.78	Plate C	D09	-7.67
**165**	Plate E	A07	-7.76	Plate B	D04	-8.76	Plate D	H08	-7.67
**166**	Plate A	A10	-7.75	Plate B	F06	-8.75	Plate A	F10	-7.66
**167**	Plate A	H10	-7.75	Plate A	A04	-8.74	Plate A	E02	-7.65
**168**	Plate C	E07	-7.75	Plate B	E10	-8.74	Plate D	B10	-7.65
**169**	Plate B	H08	-7.74	Plate D	A04	-8.73	Plate E	D09	-7.63
**170**	Plate A	A06	-7.72	Plate E	C05	-8.73	Plate A	C02	-7.62
**171**	Plate C	H06	-7.72	Plate A	C06	-8.72	Plate C	E05	-7.62
**172**	Plate E	H02	-7.72	Plate C	H05	-8.72	Plate E	C03	-7.62
**173**	Plate C	B02	-7.7	Plate E	E04	-8.72	Plate B	E03	-7.6
**174**	Plate C	E09	-7.68	Plate E	E08	-8.72	Plate C	A06	-7.6
**175**	Plate B	D09	-7.67	Plate D	F05	-8.7	Plate A	D08	-7.59
**176**	Plate D	C08	-7.67	Plate A	H10	-8.69	Plate C	D08	-7.59
**177**	Plate A	E02	-7.66	Plate C	F02	-8.69	Plate C	H10	-7.59
**178**	Plate D	F07	-7.65	Plate C	H11	-8.69	Plate E	E02	-7.56
**179**	Plate D	G06	-7.65	Plate B	B06	-8.68	Plate A	E05	-7.55
**180**	Plate B	B06	-7.64	Plate C	E07	-8.68	Plate D	F07	-7.55
**181**	Plate C	B09	-7.64	Plate C	A04	-8.67	Plate A	E03	-7.54
**182**	Plate C	C03	-7.63	Plate C	A05	-8.66	Plate D	E11	-7.54
**183**	Plate D	F04	-7.63	Plate B	G08	-8.65	Plate E	G05	-7.53
**184**	Plate E	C04	-7.62	Plate D	A07	-8.65	Plate E	G11	-7.53
**185**	Plate B	G05	-7.6	Plate A	G06	-8.64	Plate D	D11	-7.52
**186**	Plate B	H09	-7.59	Plate C	D05	-8.64	Plate B	H02	-7.5
**187**	Plate E	G10	-7.59	Plate D	B04	-8.64	Plate E	C11	-7.49
**188**	Plate A	C02	-7.57	Plate A	A03	-8.63	Plate A	G06	-7.48
**189**	Plate C	A08	-7.57	Plate A	H07	-8.63	Plate D	B03	-7.48
**190**	Plate B	E08	-7.56	Plate D	C10	-8.63	Plate C	E08	-7.47
**191**	Plate B	E10	-7.55	Plate A	E05	-8.62	Plate E	D05	-7.47
**192**	Plate C	G07	-7.55	Plate D	A03	-8.62	Plate A	G04	-7.46
**193**	Plate D	B03	-7.55	Plate E	G06	-8.62	Plate A	G09	-7.46
**194**	Plate E	B02	-7.54	Plate D	B06	-8.58	Plate B	C03	-7.46
**195**	Plate C	F07	-7.53	Plate D	G08	-8.58	Plate B	G08	-7.46
**196**	Plate A	B05	-7.52	Plate E	C11	-8.58	Plate D	C04	-7.46
**197**	Plate D	A04	-7.52	Plate B	C10	-8.57	Plate E	D10	-7.46
**198**	Plate D	A07	-7.52	Plate B	D02	-8.57	Plate E	H04	-7.46
**199**	Plate D	D11	-7.51	Plate E	C02	-8.57	Plate A	H08	-7.44
**200**	Plate C	D06	-7.5	Plate C	G07	-8.55	Plate B	G09	-7.43

Ranking of affinity	A ((3UIE)			B (4FXP)			C (2GSK)		
	Compound		Binding energy	Compound		Binding energy	Compound		Binding energy
**201**	Plate A	C08	-7.49	Plate C	H03	-8.55	Plate E	A11	-7.43
**202**	Plate E	D03	-7.49	Plate A	A10	-8.53	Plate A	B07	-7.42
**203**	Plate B	G08	-7.48	Plate B	B04	-8.53	Plate A	B11	-7.42
**204**	Plate E	G06	-7.48	Plate C	G06	-8.53	Plate E	C04	-7.41
**205**	Plate D	D03	-7.47	Plate D	C02	-8.52	Plate A	H07	-7.4
**206**	Plate E	D05	-7.47	Plate E	G11	-8.52	Plate C	H05	-7.38
**207**	Plate B	A06	-7.46	Plate B	C07	-8.51	Plate C	F02	-7.36
**208**	Plate B	B08	-7.46	Plate B	F05	-8.51	Plate C	E07	-7.35
**209**	Plate E	C02	-7.46	Plate A	E08	-8.49	Plate C	F07	-7.35
**210**	Plate D	H11	-7.45	Plate D	D11	-8.49	Plate E	G08	-7.35
**211**	Plate A	H08	-7.44	Plate D	G05	-8.49	Plate A	D03	-7.34
**212**	Plate B	F05	-7.44	Plate D	H08	-8.48	Plate D	C02	-7.33
**213**	Plate D	A06	-7.44	Plate A	F03	-8.47	Plate E	G04	-7.31
**214**	Plate D	H09	-7.4	Plate C	D07	-8.47	Plate A	B09	-7.29
**215**	Plate E	B07	-7.4	Plate D	A02	-8.47	Plate C	F11	-7.29
**216**	Plate C	E05	-7.38	Plate E	D11	-8.47	Plate C	D04	-7.28
**217**	Plate E	H04	-7.38	Plate D	G11	-8.45	Plate C	G05	-7.28
**218**	Plate C	H11	-7.37	Plate E	A07	-8.45	Plate A	G05	-7.27
**219**	Plate B	D07	-7.35	Plate A	B07	-8.44	Plate B	H08	-7.27
**220**	Plate C	C02	-7.34	Plate E	D05	-8.43	Plate E	F02	-7.27
**221**	Plate D	G11	-7.34	Plate B	A07	-8.42	Plate D	A09	-7.25
**222**	Plate A	A09	-7.32	Plate E	A10	-8.42	Plate B	C07	-7.24
**223**	Plate B	A07	-7.32	Plate E	E02	-8.41	Plate B	E10	-7.24
**224**	Plate B	D02	-7.32	Plate E	F02	-8.41	Plate A	G03	-7.23
**225**	Plate B	E04	-7.31	Plate A	B10	-8.4	Plate C	A04	-7.23
**226**	Plate D	E04	-7.31	Plate C	G09	-8.4	Plate C	D11	-7.23
**227**	Plate E	A04	-7.31	Plate A	A08	-8.38	Plate C	G09	-7.23
**228**	Plate C	B05	-7.3	Plate A	B11	-8.37	Plate C	H11	-7.23
**229**	Plate D	B05	-7.3	Plate C	G02	-8.37	Plate D	D04	-7.23
**230**	Plate E	B05	-7.3	Plate D	B11	-8.37	Plate E	A09	-7.23
**231**	Plate A	F06	-7.29	Plate A	C04	-8.34	Plate A	D06	-7.22
**232**	Plate B	H10	-7.29	Plate E	B03	-8.33	Plate B	F06	-7.22
**233**	Plate A	F09	-7.28	Plate E	H02	-8.33	Plate D	G11	-7.21
**234**	Plate B	D10	-7.28	Plate E	F03	-8.32	Plate E	D07	-7.21
**235**	Plate C	G04	-7.28	Plate E	B04	-8.31	Plate B	G06	-7.2
**236**	Plate C	G10	-7.27	Plate A	H09	-8.3	Plate A	A09	-7.19
**237**	Plate C	G02	-7.25	Plate B	A06	-8.3	Plate B	A04	-7.19
**238**	Plate D	A11	-7.25	Plate B	E03	-8.3	Plate E	B02	-7.19
**239**	Plate A	E03	-7.24	Plate A	F04	-8.29	Plate C	E10	-7.17
**240**	Plate E	F02	-7.24	Plate C	C02	-8.29	Plate D	A11	-7.17
**241**	Plate C	A03	-7.23	Plate B	G06	-8.28	Plate D	E04	-7.17
**242**	Plate C	B04	-7.23	Plate D	B05	-8.27	Plate B	G07	-7.16
**243**	Plate D	C04	-7.23	Plate B	G09	-8.23	Plate B	A06	-7.15
**244**	Plate D	H07	-7.22	Plate D	B08	-8.23	Plate C	D07	-7.15
**245**	Plate E	E11	-7.21	Plate D	F06	-8.23	Plate D	A06	-7.15
**246**	Plate B	E05	-7.2	Plate E	A03	-8.23	Plate C	A03	-7.14
**247**	Plate A	B10	-7.19	Plate D	E11	-8.22	Plate C	G10	-7.14
**248**	Plate A	B11	-7.19	Plate C	F11	-8.21	Plate C	H06	-7.13
**249**	Plate D	B11	-7.19	Plate A	A09	-8.2	Plate A	A02	-7.12
**250**	Plate D	E02	-7.19	Plate B	F02	-8.2	Plate B	F05	-7.12
**251**	Plate D	E11	-7.19	Plate C	H07	-8.2	Plate B	C09	-7.11
**252**	Plate A	E04	-7.18	Plate C	B04	-8.17	Plate B	E08	-7.1
**253**	Plate A	G11	-7.18	Plate C	D04	-8.17	Plate C	A02	-7.1
**254**	Plate A	H07	-7.18	Plate E	G05	-8.17	Plate C	G02	-7.1
**255**	Plate E	A11	-7.18	Plate A	A07	-8.16	Plate A	B05	-7.09
**256**	Plate A	F05	-7.16	Plate D	D02	-8.15	Plate B	D02	-7.09
**257**	Plate B	F02	-7.16	Plate E	A11	-8.13	Plate C	A08	-7.09
**258**	Plate C	H10	-7.16	Plate A	D03	-8.12	Plate D	B05	-7.09
**259**	Plate B	D11	-7.14	Plate B	B02	-8.1	Plate D	B06	-7.08
**260**	Plate D	A02	-7.13	Plate D	H09	-8.1	Plate C	A05	-7.07
**261**	Plate A	G03	-7.12	Plate D	F07	-8.09	Plate C	B04	-7.07
**262**	Plate C	A04	-7.12	Plate E	A06	-8.08	Plate B	B03	-7.06
**263**	Plate C	G11	-7.11	Plate D	F11	-8.07	Plate B	C08	-7.06
**264**	Plate C	H07	-7.1	Plate E	B10	-8.05	Plate D	H11	-7.06
**265**	Plate A	D06	-7.09	Plate A	G03	-8.04	Plate E	F05	-7.06
**266**	Plate D	G10	-7.09	Plate C	C04	-8.03	Plate A	H10	-7.04
**267**	Plate E	B03	-7.09	Plate A	D06	-8.02	Plate D	D02	-7.04
**268**	Plate A	A08	-7.08	Plate B	E08	-8.02	Plate C	D02	-7.03
**269**	Plate A	D10	-7.08	Plate A	D08	-8.01	Plate C	D03	-7.03
**270**	Plate E	B06	-7.08	Plate E	D10	-7.99	Plate E	B08	-7.03
**271**	Plate E	E06	-7.07	Plate D	E08	-7.97	Plate E	D11	-7.03
**272**	Plate A	C11	-7.06	Plate D	G03	-7.97	Plate A	E07	-7.02
**273**	Plate D	H06	-7.06	Plate A	A02	-7.96	Plate C	E04	-7.02
**274**	Plate E	D09	-7.06	Plate B	D06	-7.96	Plate A	B04	-7.01
**275**	Plate B	B02	-7.05	Plate C	E04	-7.96	Plate E	C02	-7.01
**276**	Plate E	F08	-7.05	Plate A	B05	-7.94	Plate D	G05	-7
**277**	Plate A	E08	-7.04	Plate D	D05	-7.93	Plate A	A10	-6.99
**278**	Plate C	F11	-7.04	Plate A	E02	-7.9	Plate E	B04	-6.98
**279**	Plate E	G05	-7.04	Plate A	H04	-7.89	Plate C	D06	-6.97
**280**	Plate C	G09	-7.01	Plate B	H09	-7.89	Plate C	H02	-6.97
**281**	Plate E	F03	-7.01	Plate C	B06	-7.88	Plate A	D11	-6.95
**282**	Plate E	C08	-6.99	Plate A	C09	-7.87	Plate C	D05	-6.94
**283**	Plate A	D07	-6.98	Plate C	G10	-7.87	Plate C	F05	-6.93
**284**	Plate A	D09	-6.98	Plate C	G05	-7.86	Plate A	H04	-6.92
**285**	Plate A	H04	-6.98	Plate E	E11	-7.85	Plate B	H10	-6.92
**286**	Plate C	D05	-6.98	Plate E	E06	-7.82	Plate D	E08	-6.91
**287**	Plate D	G02	-6.98	Plate A	C11	-7.81	Plate E	E08	-6.9
**288**	Plate A	B04	-6.96	Plate C	A02	-7.81	Plate A	E08	-6.89
**289**	Plate C	C07	-6.95	Plate B	B08	-7.8	Plate B	A03	-6.89
**290**	Plate D	A03	-6.95	Plate D	H06	-7.8	Plate D	C10	-6.89
**291**	Plate B	G06	-6.94	Plate E	F05	-7.8	Plate E	F03	-6.89
**292**	Plate D	D04	-6.94	Plate E	E07	-7.79	Plate E	G06	-6.89
**293**	Plate B	C06	-6.93	Plate D	G02	-7.78	Plate A	H09	-6.88
**294**	Plate B	F08	-6.93	Plate A	E06	-7.77	Plate C	G11	-6.88
**295**	Plate B	G09	-6.89	Plate C	A03	-7.77	Plate D	E02	-6.87
**296**	Plate C	A09	-6.89	Plate D	H11	-7.77	Plate A	E06	-6.86
**297**	Plate C	C11	-6.89	Plate E	B02	-7.76	Plate C	C02	-6.86
**298**	Plate C	A07	-6.88	Plate E	H10	-7.75	Plate C	G04	-6.85
**299**	Plate C	D09	-6.88	Plate C	C07	-7.74	Plate D	F06	-6.84
**300**	Plate A	D11	-6.87	Plate E	C09	-7.74	Plate D	E03	-6.83

Ranking of affinity	A ((3UIE)			B (4FXP)			C (2GSK)		
	Compound		Binding energy	Compound		Binding energy	Compound		Binding energy
**301**	Plate C	F05	-6.84	Plate E	D04	-7.74	Plate D	E09	-6.83
**302**	Plate D	F06	-6.84	Plate D	G06	-7.73	Plate E	H02	-6.83
**303**	Plate C	D11	-6.83	Plate B	F08	-7.72	Plate A	D09	-6.8
**304**	Plate D	C02	-6.83	Plate A	D09	-7.71	Plate A	E10	-6.8
**305**	Plate D	F03	-6.83	Plate B	D11	-7.71	Plate A	C03	-6.79
**306**	Plate E	H10	-6.83	Plate D	E03	-7.71	Plate D	H07	-6.79
**307**	Plate C	E04	-6.82	Plate D	E09	-7.71	Plate B	B02	-6.78
**308**	Plate E	F09	-6.81	Plate A	D11	-7.7	Plate D	G03	-6.78
**309**	Plate B	E03	-6.8	Plate C	G11	-7.7	Plate E	B07	-6.78
**310**	Plate D	D02	-6.79	Plate C	B08	-7.69	Plate E	F08	-6.78
**311**	Plate B	B03	-6.77	Plate D	E04	-7.68	Plate D	A03	-6.77
**312**	Plate E	A03	-6.77	Plate C	D02	-7.67	Plate A	D10	-6.76
**313**	Plate A	H09	-6.76	Plate D	D04	-7.65	Plate C	H07	-6.76
**314**	Plate B	G07	-6.73	Plate D	A06	-7.64	Plate E	E05	-6.76
**315**	Plate C	D03	-6.73	Plate E	D02	-7.64	Plate E	B03	-6.75
**316**	Plate E	E04	-6.73	Plate B	H04	-7.63	Plate B	H04	-6.74
**317**	Plate C	C04	-6.71	Plate B	G11	-7.61	Plate C	A07	-6.74
**318**	Plate E	D02	-6.71	Plate C	D03	-7.61	Plate E	E09	-6.73
**319**	Plate A	C07	-6.69	Plate B	H02	-7.6	Plate E	G10	-6.71
**320**	Plate D	D05	-6.69	Plate E	B06	-7.6	Plate B	D07	-6.69
**321**	Plate C	F08	-6.68	Plate B	H08	-7.59	Plate E	E04	-6.69
**322**	Plate D	E06	-6.68	Plate E	G10	-7.59	Plate A	B10	-6.68
**323**	Plate E	F05	-6.68	Plate E	D09	-7.58	Plate E	C09	-6.67
**324**	Plate A	E10	-6.67	Plate A	E10	-7.57	Plate C	C04	-6.65
**325**	Plate B	G11	-6.67	Plate C	C11	-7.57	Plate D	A10	-6.65
**326**	Plate B	H04	-6.67	Plate C	G08	-7.57	Plate E	D02	-6.65
**327**	Plate C	E10	-6.67	Plate E	B08	-7.55	Plate C	F10	-6.63
**328**	Plate C	G05	-6.67	Plate C	E05	-7.52	Plate D	D05	-6.63
**329**	Plate E	C09	-6.67	Plate A	E04	-7.49	Plate E	H05	-6.62
**330**	Plate E	H11	-6.67	Plate D	H07	-7.46	Plate E	B06	-6.61
**331**	Plate C	G03	-6.66	Plate A	C10	-7.44	Plate A	C11	-6.6
**332**	Plate B	D04	-6.65	Plate A	G09	-7.41	Plate B	F07	-6.59
**333**	Plate D	E08	-6.65	Plate D	E02	-7.41	Plate A	C09	-6.58
**334**	Plate D	H05	-6.62	Plate C	E10	-7.37	Plate C	B06	-6.58
**335**	Plate E	A08	-6.61	Plate E	F08	-7.37	Plate D	B02	-6.58
**336**	Plate E	G11	-6.61	Plate A	F05	-7.34	Plate D	G06	-6.58
**337**	Plate A	G09	-6.6	Plate A	C08	-7.33	Plate D	G02	-6.57
**338**	Plate D	B08	-6.58	Plate D	B02	-7.31	Plate A	C08	-6.56
**339**	Plate D	E03	-6.56	Plate B	A03	-7.27	Plate B	G10	-6.53
**340**	Plate C	A06	-6.53	Plate D	F03	-7.27	Plate E	F11	-6.53
**341**	Plate D	E09	-6.53	Plate E	F09	-7.25	Plate E	H06	-6.53
**342**	Plate A	G10	-6.52	Plate E	D07	-7.24	Plate E	E06	-6.51
**343**	Plate E	A06	-6.52	Plate A	G10	-7.23	Plate A	C04	-6.48
**344**	Plate C	G08	-6.51	Plate C	D11	-7.23	Plate D	F03	-6.48
**345**	Plate D	G05	-6.51	Plate E	A05	-7.23	Plate A	F05	-6.46
**346**	Plate E	E08	-6.51	Plate A	G08	-7.22	Plate C	G03	-6.44
**347**	Plate A	B03	-6.5	Plate B	F03	-7.22	Plate A	A08	-6.42
**348**	Plate B	H05	-6.48	Plate C	B07	-7.21	Plate B	D04	-6.42
**349**	Plate D	G03	-6.48	Plate A	C07	-7.19	Plate D	E06	-6.42
**350**	Plate E	E07	-6.46	Plate B	D07	-7.19	Plate D	F04	-6.42
**351**	Plate A	C03	-6.45	Plate C	A07	-7.12	Plate A	C10	-6.41
**352**	Plate C	D02	-6.43	Plate A	D10	-7.1	Plate C	G08	-6.4
**353**	Plate E	B08	-6.41	Plate A	G04	-7.08	Plate A	G10	-6.39
**354**	Plate E	G07	-6.41	Plate E	G03	-7.08	Plate E	F09	-6.39
**355**	Plate A	A02	-6.4	Plate C	A06	-7.06	Plate B	F08	-6.36
**356**	Plate C	C06	-6.36	Plate B	F07	-7.02	Plate B	D06	-6.31
**357**	Plate D	B02	-6.36	Plate D	F04	-7.02	Plate A	C07	-6.28
**358**	Plate E	B04	-6.36	Plate E	E09	-7.02	Plate C	G06	-6.28
**359**	Plate E	A05	-6.34	Plate C	G03	-6.99	Plate E	E11	-6.28
**360**	Plate E	H09	-6.34	Plate C	H10	-6.98	Plate E	H11	-6.27
**361**	Plate A	F08	-6.33	Plate E	A08	-6.96	Plate A	B02	-6.26
**362**	Plate A	E06	-6.32	Plate A	A05	-6.94	Plate A	D07	-6.23
**363**	Plate C	F10	-6.25	Plate A	B02	-6.92	Plate E	E07	-6.2
**364**	Plate E	G03	-6.25	Plate B	G07	-6.92	Plate B	D05	-6.17
**365**	Plate C	B06	-6.24	Plate A	D07	-6.91	Plate C	A09	-6.14
**366**	Plate E	D07	-6.21	Plate C	A09	-6.91	Plate B	E04	-6.11
**367**	Plate E	D04	-6.19	Plate D	H05	-6.88	Plate C	C11	-6.11
**368**	Plate C	A02	-6.15	Plate A	C03	-6.86	Plate D	H05	-6.07
**369**	Plate C	B07	-6.15	Plate B	H05	-6.84	Plate E	H09	-6.02
**370**	Plate C	G06	-6.15	Plate A	F08	-6.79	Plate A	F08	-5.99
**371**	Plate E	H06	-6.15	Plate C	C06	-6.74	Plate B	E06	-5.99
**372**	Plate A	C09	-6.13	Plate B	E06	-6.7	Plate E	A08	-5.99
**373**	Plate A	G08	-6.11	Plate B	D05	-6.69	Plate A	G08	-5.94
**374**	Plate B	E06	-6.04	Plate E	H09	-6.69	Plate C	B07	-5.93
**375**	Plate B	F07	-6.01	Plate B	E04	-6.66	Plate E	A03	-5.92
**376**	Plate B	A04	-5.85	Plate A	B03	-6.65	Plate A	B03	-5.91
**377**	Plate E	C10	-5.83	Plate E	H11	-6.65	Plate A	E04	-5.87
**378**	Plate A	B02	-5.78	Plate E	H06	-6.5	Plate E	D04	-5.87
**379**	Plate B	D05	-5.71	Plate B	C05	-6.49	Plate B	H05	-5.86
**380**	Plate B	G10	-5.71	Plate D	E06	-6.49	Plate C	C06	-5.84
**381**	Plate B	D06	-5.68	Plate E	G07	-6.46	Plate B	A05	-5.78
**382**	Plate C	H08	-5.62	Plate C	F10	-6.43	Plate C	B08	-5.77
**383**	Plate B	A05	-5.56	Plate C	F08	-6.42	Plate B	G11	-5.7
**384**	Plate D	F11	-5.55	Plate B	G10	-6.38	Plate C	H08	-5.65
**385**	Plate B	A03	-5.54	Plate C	H08	-6.29	Plate D	F11	-5.64
**386**	Plate C	B08	-5.5	Plate B	A05	-6.28	Plate B	H03	-5.63
**387**	Plate E	E09	-5.49	Plate E	B05	-6.25	Plate C	F08	-5.63
**388**	Plate E	H08	-5.41	Plate E	C10	-6.25	Plate E	C10	-5.51
**389**	Plate E	B09	-5.38	Plate E	F11	-6.24	Plate E	G07	-5.31
**390**	Plate B	H03	-5.14	Plate B	H03	-6.12	Plate E	H08	-5.2
**391**	Plate E	H05	-5.13	Plate E	H08	-6.12	Plate E	G03	-5.17
**392**	Plate B	F04	-4.87	Plate E	H05	-6.06	Plate E	B09	-5.12
**393**	Plate E	A02	-4.8	Plate B	G02	-5.7	Plate B	F04	-5.02
**394**	Plate B	G02	-4.79	Plate E	A02	-5.47	Plate E	A02	-5.01
**395**	Plate E	H03	-4.79	Plate E	B09	-5.32	Plate E	E03	-4.61
**396**	Plate B	C05	-4.73	Plate B	F04	-4.98	Plate B	G02	-4.41
**397**	Plate E	F11	-4.18	Plate B	G04	-4.32	Plate B	H11	-4.07
**398**	Plate E	E03	-3.85	Plate E	E03	-4.26	Plate B	C05	-3.97
**399**	Plate B	G04	-3.79	Plate B	H11	-3.28	Plate B	G04	-2.93
**400**	Plate B	H11	-3.77	Plate E	H03	-2.24	Plate E	H03	-1.57
