# Supplementary material for: Characterization of Entamoeba histolytica adenosine 5′-phosphosulfate (APS) kinase; validation as a target and provision of leads for the development of new drugs against amoebiasis
Source: PLoS Negl Trop Dis. 2019 Aug 19;13(8):e0007633. doi: 10.1371/journal.pntd.0007633 (PMC6715247; doi:10.1371/journal.pntd.0007633)
Supplement: S3 Table — Ranking of in vitro data for EhAPSK and HsAPSK is based on inhibition levels against rEhAPSK (from Table 2) and rHsAPSK (from S4 Fig) activities, respectively. Ranking of in silico data are based on the binding energies determined by computer simulated docking analysis between either EhAPSK structure-A or the tertiary structure of HsPAPSS1 (PDB ID, 1XNJ) and each compound in the Pathogen Box. NS, not significantly inhibited. (PDF) [file pntd.0007633.s003.pdf]

Pathogen Box			Ranking	A (3UIE)		Ranking	HsPAPSS1 (1XNJ)	
Rack	Position	Trivial name	inhibition level of rEhAPSK	Ranking (*in*	Binding energy	inhibition level of rHsAPSK	Ranking (*in*	Binding energy
Plate D	E10		**1**	**13**	-9.5	**NS**	42	-9.47
Plate B	D03	suramin	**2**	**2**	-11.11	**NS**	**4**	-10.87
Plate E	H05	auranofin	**3**	391	-5.13	**NS**	320	-7.16
Plate C	F03		**4**	49	-8.87	**NS**	57	-9.2
Plate B	C02		**5**	**3**	-10.34	**NS**	**6**	-10.67
Plate A	C05		**6**	**1**	-11.36	**NS**	**2**	-11.39
Plate B	B05		**7**	56	-8.82	**NS**	90	-8.82
Plate A	D11		**8**	300	-6.87	**NS**	212	-7.97
Plate B	C08		**9**	**14**	-9.43	**NS**	92	-8.79
Plate A	H11		**10**	105	-8.21	**NS**	168	-8.24
Plate A	F07		**11**	58	-8.8	**NS**	61	-9.13
Plate C	C06		**12**	356	-6.36	**NS**	355	-6.6
Plate A	F04		**13**	137	-7.94	**NS**	129	-8.48
Plate C	F06	delamanid	**13**	**4**	-10.28	**NS**	**16**	-10
Plate E	G10		**13**	187	-7.59	**NS**	263	-7.6
