# Supplementary material for: Characterization of Entamoeba histolytica adenosine 5′-phosphosulfate (APS) kinase; validation as a target and provision of leads for the development of new drugs against amoebiasis
Source: PLoS Negl Trop Dis. 2019 Aug 19;13(8):e0007633. doi: 10.1371/journal.pntd.0007633 (PMC6715247; doi:10.1371/journal.pntd.0007633)
Supplement: S4 Table — (PDF) [file pntd.0007633.s004.pdf]

Pathogen Box		Compound ID in MMV	Disease set[a]	Susceptible organisms[b]	reference
Compound	Trivial name				
D-E-10		MMV688271	Trypanosomiasis and Leishmaniasis	*Burkholderia pseudomallei*	[44]
				Cryptococcus neoformans	[48]
				Candida albicans	[48]
				Trypanosoma brucei brucei	[42]
				Plasmodium falciparum	[49]
B-D-03	suramin	MMV637953	Trypanosomiasis, Onchocerciasis		
E-H-05	auranofin	MMV688978	Amebiasis, Rheumatoid arthritis	*Entamoeba histolytica*	[18], [19]
				Giardia intestinalis	[45]
				Cryptosporidium parvum	[45]
C-F-03		MMV688179	Trypanosomiasis and Leishmaniasis	*Burkholderia pseudomallei*	[44]
				Plasmodium falciparum	[49]
B-C-02		MMV690027	Trypanosomiasis and Leishmaniasis	*Trypanosoma brucei brucei*	[42]
A-C-05		MMV690028	Trypanosomiasis and Leishmaniasis	*Trypanosoma brucei brucei*	[42]
B-B-05		MMV687776	Onchocerciasis	*Trypanosoma brucei brucei*	[42]
				Trypanosoma cruzi	[49]
A-D-11		MMV676409	Tuberculosis	*Trypanosoma brucei brucei*	[42]
B-C-08		MMV020710	Malaria	*Plasmodium falciparum*	[46]
A-H-11		MMV676512	Tuberculosis	*Toxoplasma gondii*	[43]
				Trypanosoma brucei brucei	[42]
A-F-07		MMV676476	Tuberculosis		
C-C-06		MMV687251	Tuberculosis	*Staphylococcus aureus*	[47]
A-F-04		MMV676388	Tuberculosis	*Plasmodium falciparum*	[49]
C-F-06	delamanid	MMV688262	Tuberculosis	*Giardia intestinalis*	[45]
E-G-10		MMV688263	Onchocerciasis		

[a]Described in detail on the MMV website (https://www.mmv.org/mmv-open/pathogen-box/about-pathogen-box).
[b]Data are contained in the References cited in the next column.
