# Supplementary figures and images for: Characterization of Entamoeba histolytica adenosine 5′-phosphosulfate (APS) kinase; validation as a target and provision of leads for the development of new drugs against amoebiasis
Source: PLoS Negl Trop Dis. 2019 Aug 19;13(8):e0007633. doi: 10.1371/journal.pntd.0007633 (PMC6715247; doi:10.1371/journal.pntd.0007633)

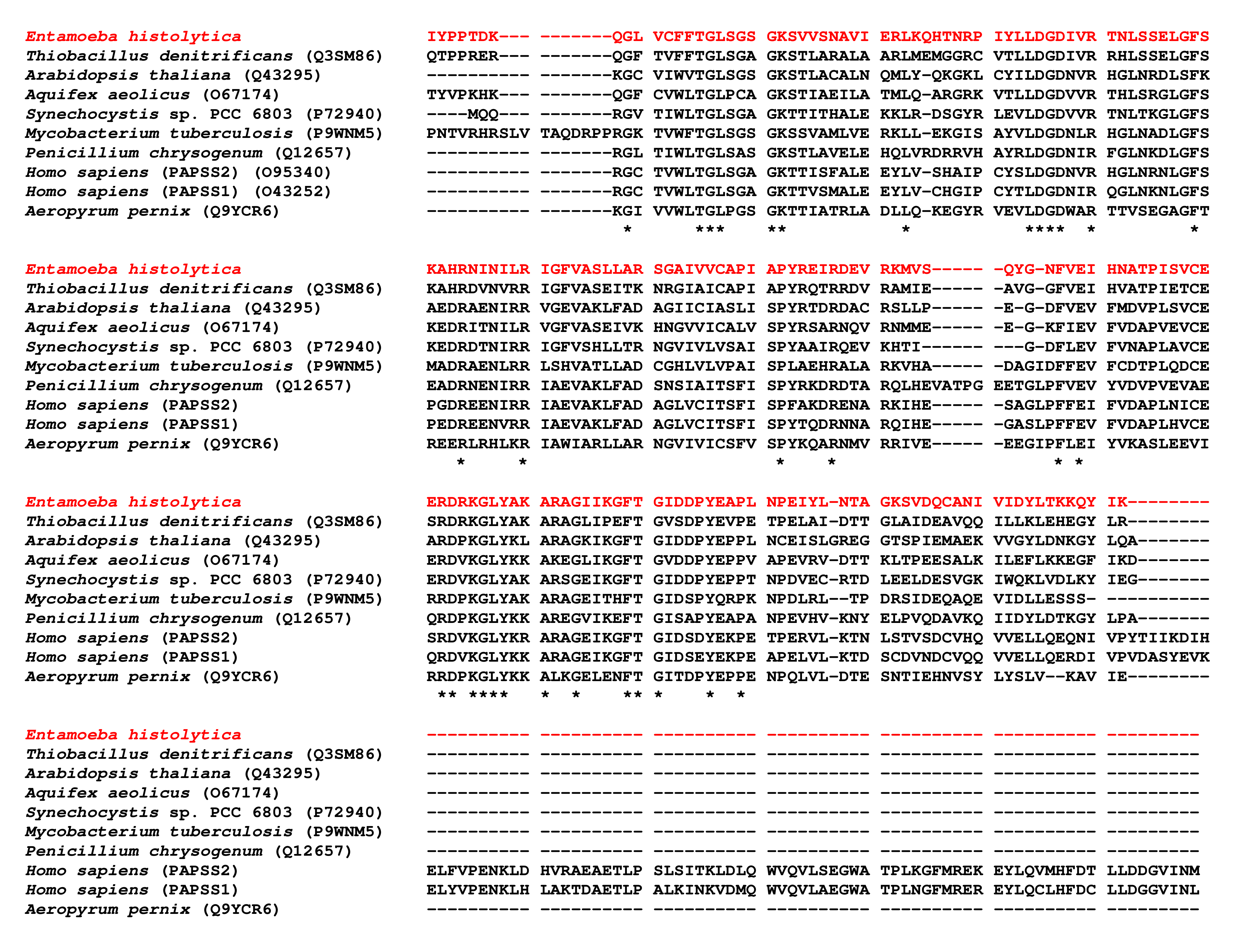

Supplement: S1 Fig — The UniProtKB ID number for each protein is indicated inside parentheses. The amino acid residues conserved in all proteins are indicated by *. (TIF) [file pntd.0007633.s005.tif]

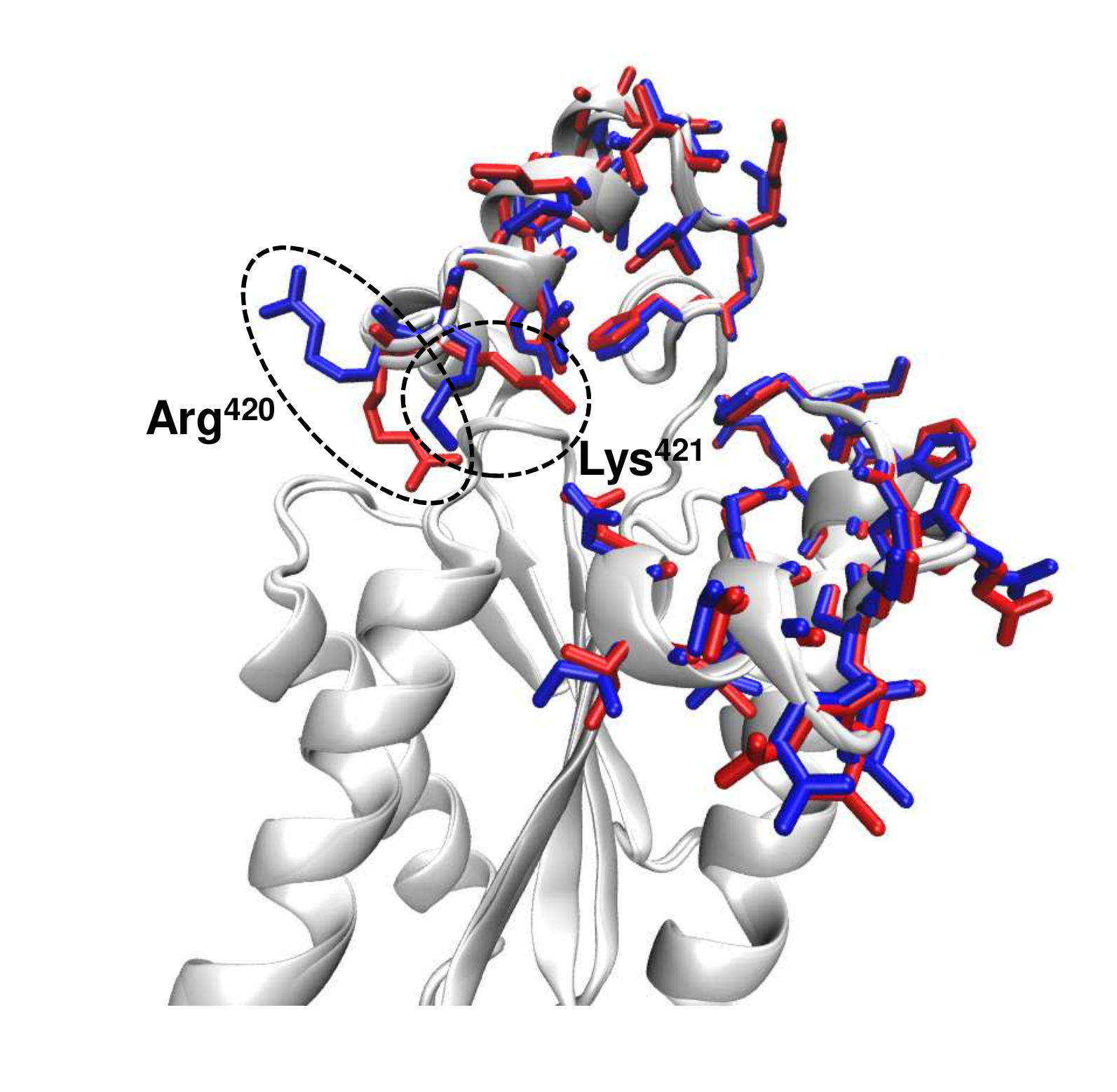

Supplement: S2 Fig — Close up of the spatial positions of side-chains in the cavity of homology-modeled EhAPSK structure-A (blue) and -B (red). (TIF) [file pntd.0007633.s006.tif]

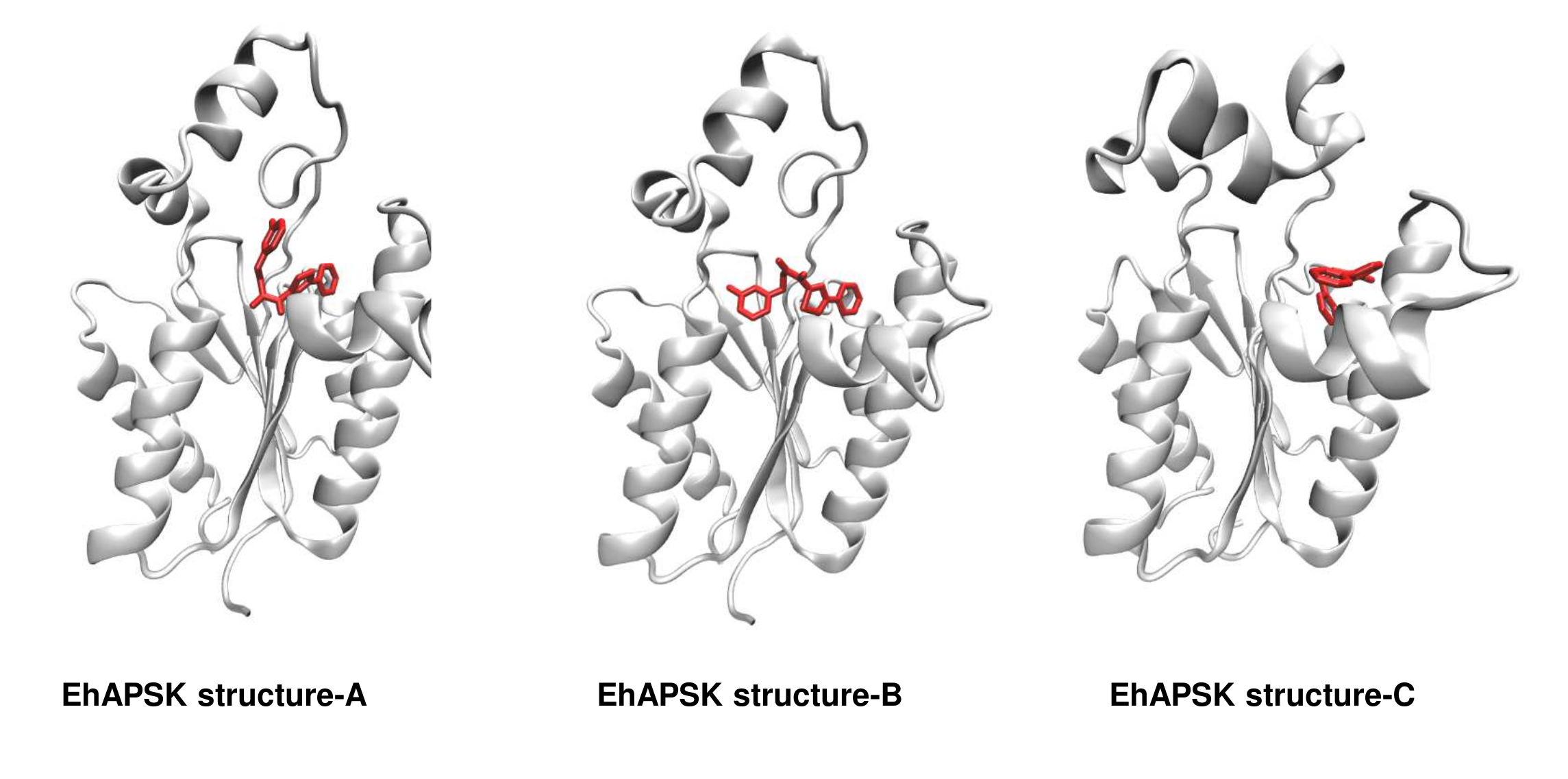

Supplement: S3 Fig — Binding pattern of A-D-11 to EhAPSK-C, which is structurally different from both EhAPSK-A and -B, is also shown for ease of comparison. A-D-11 positioned in each cavity of the homology modeled EhAPSKs is highlighted in red. (TIF) [file pntd.0007633.s007.tif]

(A)

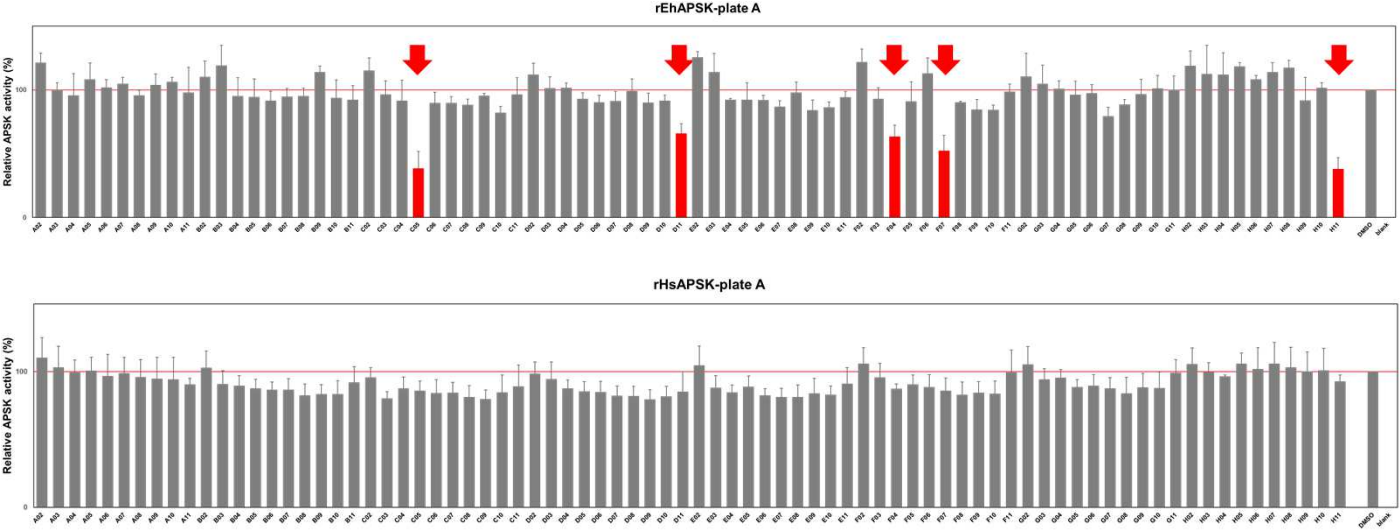

(B)

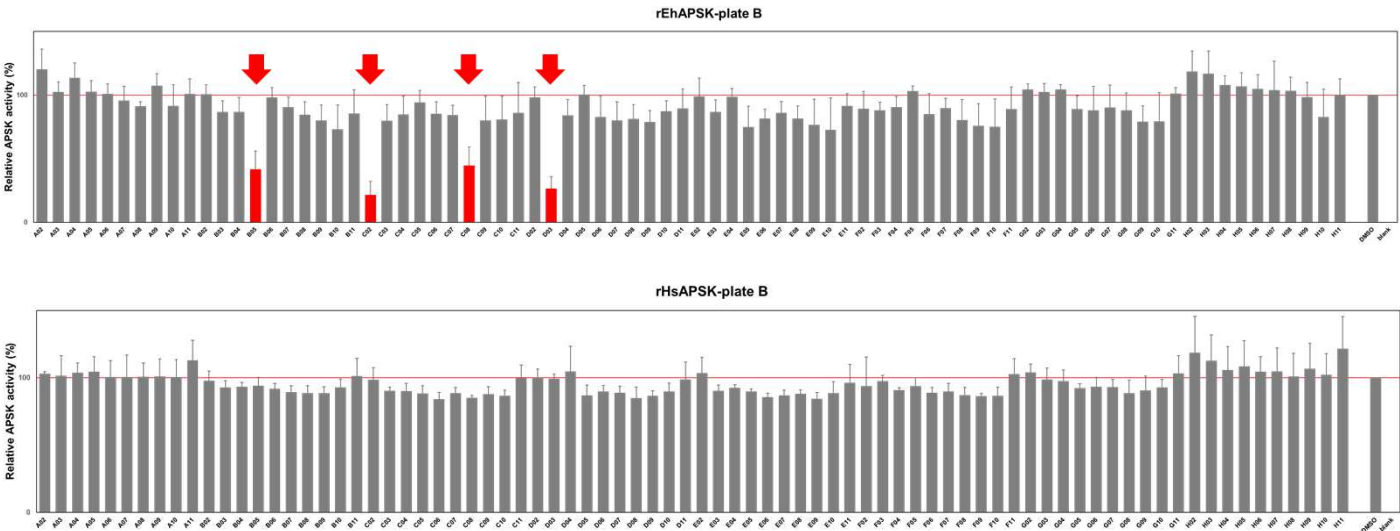

(C)

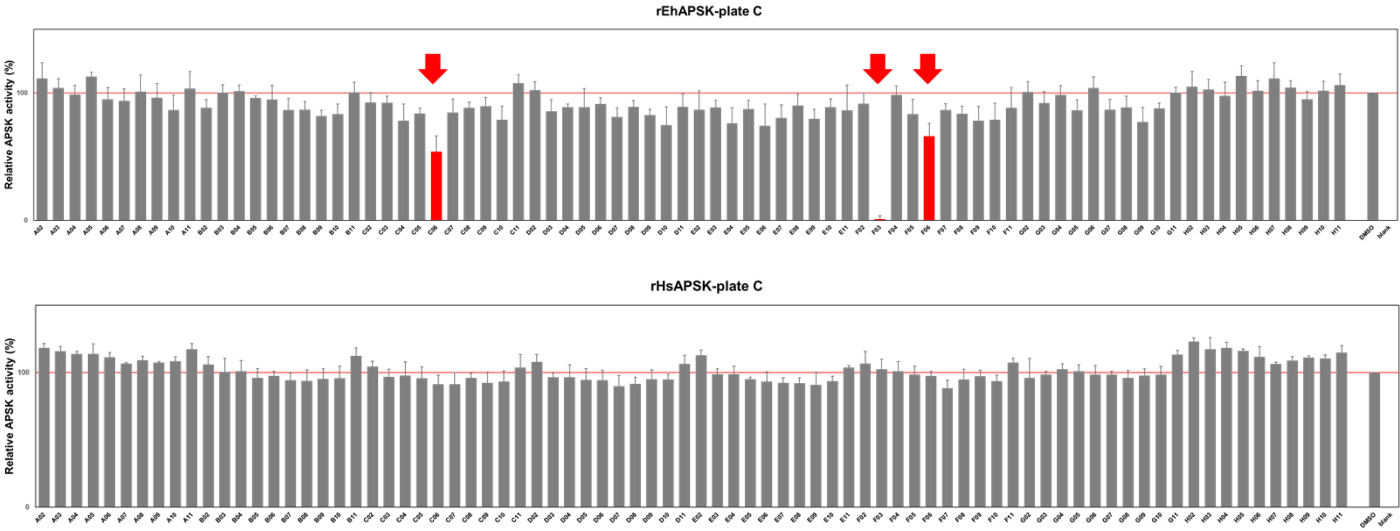

(D)

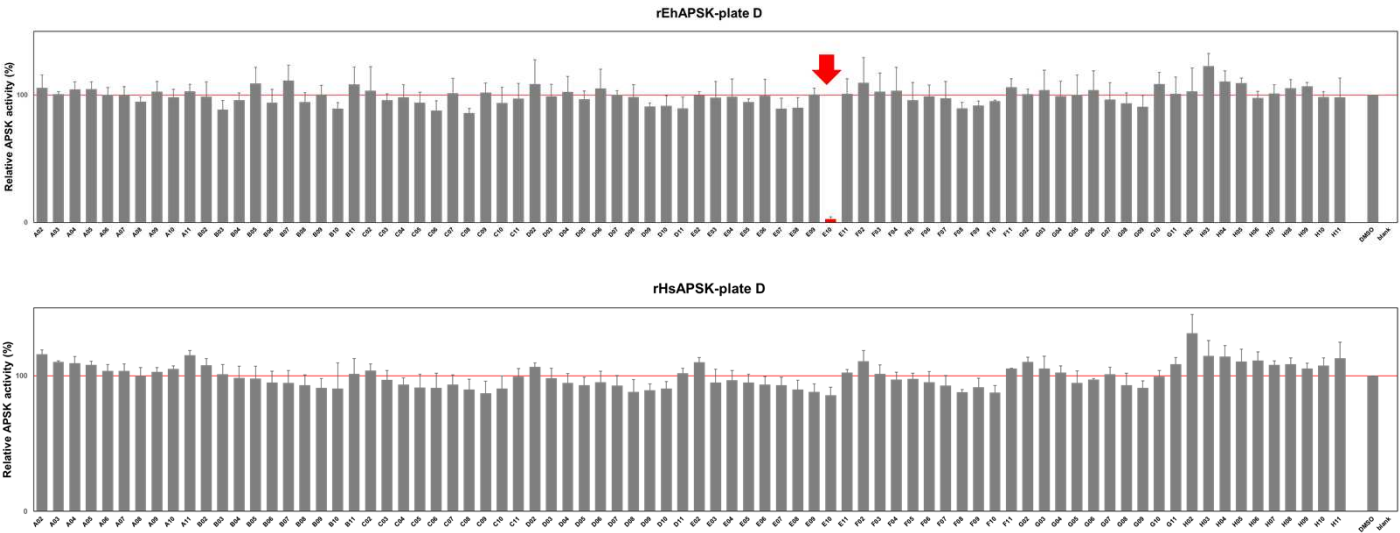

(E)

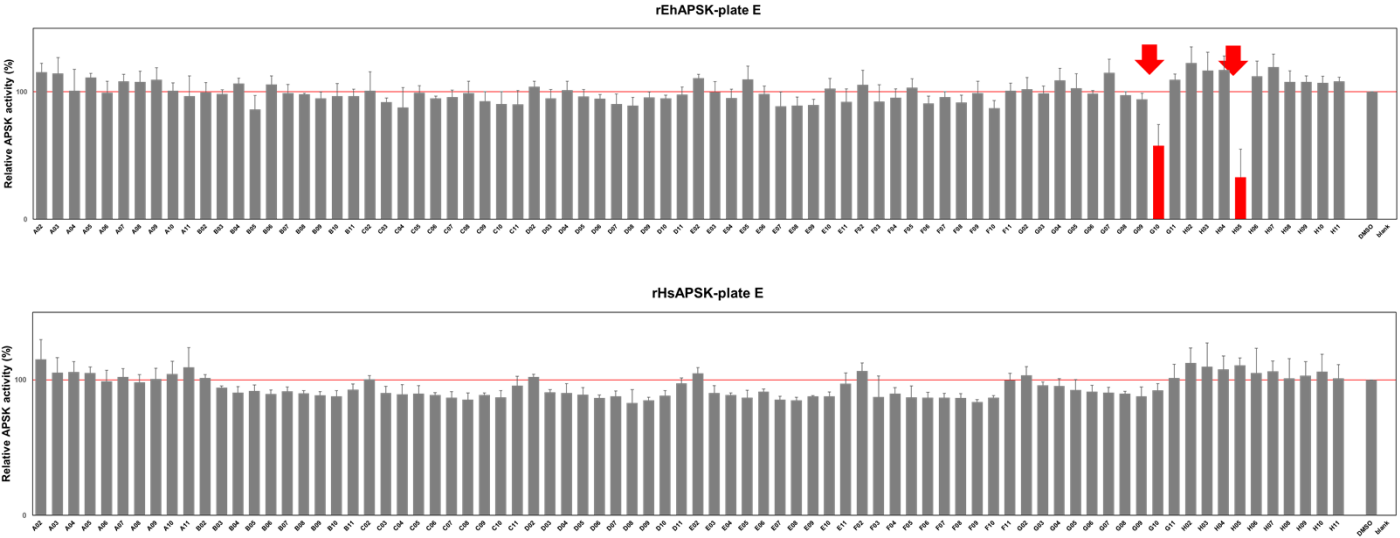

Supplement: S4 Fig — (A-E) The effect(s) on activities of rEhAPSK (upper) and rHsAPSK (lower). Data are expressed as the residual activity expressed as the percentage of the activity in each sample relative to that in DMSO control (set as 100%). Data shown are the mean with error bar (SD from the mean) from three independent experiments. Red bars and arrows indicate compounds that reproducibly inhibited rEhAPSK activity. DMSO and blank controls were included. Five 96-well plates (plate A-E) were used, into which 400 compounds together with DMSO and blank controls were equally dispensed (80 wells per plate). (PDF) [file pntd.0007633.s008.pdf]

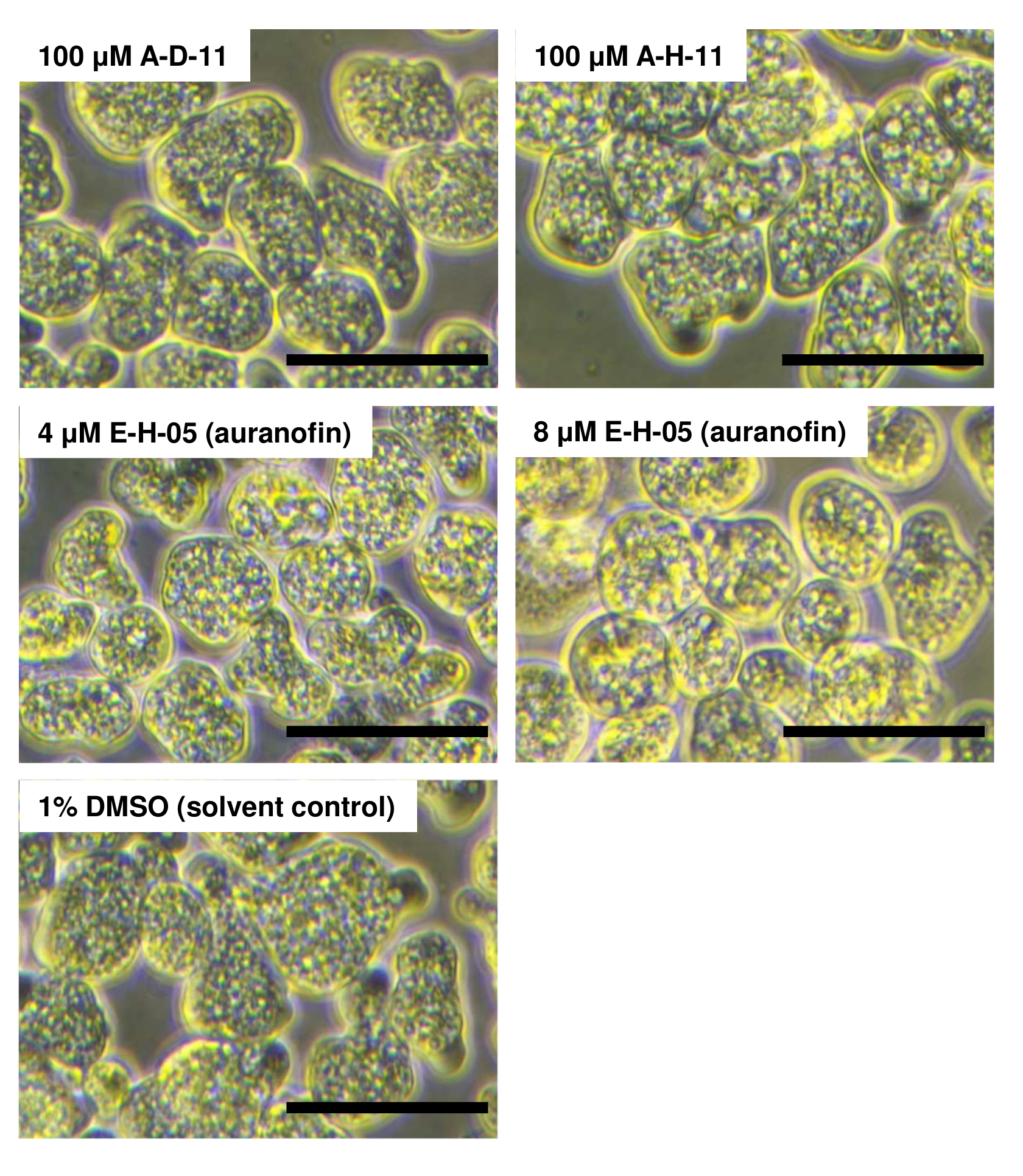

Supplement: S5 Fig — Phase contrast images of cells are shown that were treated for 6 h with A-D-11, A-H-11, or E-H-05 (auranofin) at the indicated concentrations. Bar indicates 50 μm. Representative images from three independent experiments are shown. (TIF) [file pntd.0007633.s009.tif]
